# Chemerin-induced macrophages pyroptosis in fetal brain tissue leads to cognitive disorder in offspring of diabetic dams

**DOI:** 10.1186/s12974-019-1573-6

**Published:** 2019-11-16

**Authors:** Zhaoxia Liang, Luyang Han, Dianjianyi Sun, Yanmin Chen, Qi Wu, Lixia Zhang, Menglin Zhou, Danqing Chen

**Affiliations:** 1 Department of Obstetrics, Women’s Hospital, Zhejiang University School of Medicine, Xueshi Rd #1, Hangzhou, 310006 China; 2 Key Laboratory of Reproductive Genetics (Ministry of Education), Hangzhou, Zhejiang Province China; 3 Department of Epidemiology, School of Public Health and Tropical Medicine, Tulane University, New Orleans, LA USA

**Keywords:** Chemerin, Diabetes in pregnancy, ChemR23, CCRL2, Macrophages, Pyroptosis

## Abstract

**Background:**

Chemerin is highly expressed in the serum, placenta tissue, and umbilical cord blood of diabetic mother; however, the impact of chemerin on cognitive disorders of offspring from mothers with diabetes in pregnancy remains unclear.

**Methods:**

A diabetic phenotype in pregnant mice dams was induced by streptozocin (STZ) injection or intraperitoneal injection of chemerin. Behavioral changes in offspring of diabetic dams and nondiabetic controls were assessed, and changes in chemerin, two receptors of chemerin [chemerin receptor 23 (ChemR23) and chemokine (C-C motif) receptor-like 2 (CCRL2)], macrophages, and neurons in the brain tissue were studied to reveal the underlying mechanism of the behavioral changes.

**Results:**

Chemerin treatment mimicked the STZ-induced symptom of maternal diabetes in mice along with the altered behavior of offspring in the open field test (OFT) assay. In the exploring process for potential mechanism, the brain tissues of offspring from chemerin-treated dams were observed with an increase level of macrophage infiltration and a decrease number of neuron cells. Moreover, an increased level of NOD-like receptor family pyrin domain containing 3 (NLRP3) and apoptosis-associated speck-like (Asc) protein as well as pyroptosis [characterized by increased active caspase-1 content and secretion of cytokines such as interleukin (IL) 1 beta (IL-1β) and IL-18] more activated in macrophages is also observed in the brain of these diabetic dam’s offspring, in the presence of ChemR23. In vitro, it was found that pyroptosis activation was increased in macrophages separated from the abdominal cavity of normal mice, after chemerin treatment. However, depletion of CCRL2 decreased the level of chemerin in the brain tissues of diabetic dams’ offspring; depletion of ChemR23 decreased macrophage pyroptosis, and depletion of either receptor reversed chemerin-mediated neurodevelopmental deficits and cognitive impairment of offspring of diabetic pregnant dams.

**Conclusions:**

Chemerin induced diabetic pregnant disease and CCRL2 were required to enrich chemerin in the brain of offspring. Aggregation of chemerin could lead to macrophage recruitment, activation of pyroptosis, the release of inflammatory cytokines, a decrease in the number of neurons, and cognitive impairment in offspring in a ChemR23-dependent manner. Targeting CCRL2 and/or ChemR23 could be useful for treating neuropsychological deficits in offspring of dams with diabetes in pregnancy.

**Electronic supplementary material:**

The online version of this article (10.1186/s12974-019-1573-6) contains supplementary material, which is available to authorized users.

## Background

Diabetes or hyperglycemia is very common during pregnancy, with 21.3 million live births (16.2%) estimated to be affected by some form of hyperglycemia in pregnancy in a year around the world [1]. Maternal diabetes is able to produce an adverse in utero environment that may damage the embryonic development, leading to subsequent increased risk for future disease [2]. A dysfunction of glucose metabolism in pregnancy can produce short-term metabolic problems for the offspring, including macrosomia, as well as long-term problems such as cardiometabolic disorders, which manifest later in life [3]. Epidemiological studies indicate that diabetic pregnancy can also lead to neuropsychological deficits in offspring, such as lower general intelligence, attention deficit, and psychological or behavioral problems [4–6]. However, the underlying mechanism of these fetal and neonatal complications, such as neuropsychological deficits, derived from diabetic mothers remains unclear.

Abnormal neurodevelopment of offspring may be caused by inflammation. Pro-inflammatory cytokines are widely reported to suppress proliferation of neural progenitor cells, and high levels of pro-inflammatory factors can induce apoptosis in neonatal neurons [7]. Tian et al. reported that tumor necrosis factor-α and interleukin (IL)-6 levels in cord blood are excellent diagnostic indicators for brain damage in neonates with non-asphyxia fetal distress [8].

Emerging experimental evidence indicates that inflammatory damage to the brain can result from diabetic disease in pregnancy, although the mechanistic details are lacking [9, 10]. Chemerin, a newly discovered adipocytokine, is involved in metabolic diseases and regulation of inflammation [11]. Our previous research and other similar studies have shown that patients in the process of diabetic pregnancy have a higher level of chemerin in the blood, placental tissue, and cord blood [12–14]. On the other hand, chemerin and its receptor, chemerin receptor 23 (ChemR23), play a key role in ChemR23^+^ leukocyte (like macrophages) infiltration of the central nervous system and the development of autoimmune demyelinating disease [15]; chemerin is an inducer of the NOD-like receptor family pyrin domain containing 3 (NLRP3) inflammasome in macrophages residing in the liver [16].

Therefore, we speculate that a high level of chemerin in women with diabetic pregnancy might cause ChemR23^+^ leukocyte-related inflammation in the fetal brain, affecting a cognitive disorder in the offspring. Specifically, we hypothesize that the level of chemerin in the fetal brain increases to chemotactically recruit ChemR23^+^ cells, causing NLRP3-related inflammatory brain injury and neuropsychological deficits in offspring.

## Methods and material

### Reagents and antibodies

The reagents and antibodies were as follows: chemerin (2325-CM; R&D Systems, Minneapolis, MN, USA), Transwell chambers (3422; Corning Inc., Corning, NY, USA), MiniBEST Universal RNA Extraction Kit (9767, Takara; Shiga, Japan), PrimeScript® RT reagent Kit with gDNA Eraser (RR047; Takara), qPCR Kit (SYBR Premix Ex Taq) (638319; Takara), anti-chemerin (ab103153; Abcam, Cambridge, UK), anti-ChemR23 (ab64881; Abcam), anti-chemokine (C-C motif) receptor-like 2 (CCRL2) (ab88632; Abcam), anti-NLRP3 (ab214185; Abcam), anti-Asc (ab175449; Abcam), anti-caspase-1 (24232, 89332; Cellular Signaling Technology, Danvers, MA, USA), anti-caspase-3 (9662; Cellular Signaling Technology), anti-caspase-7 (12827; Cellular Signaling Technology), anti-caspase-8 (8592; Cellular Signaling Technology), anti-IL-1β (MM425B, PA5-95455; Thermo Fisher, Waltham, MA, USA), anti-IL-18 (04-1585; Millipore, Bedford, MA, USA), anti-glyceraldehyde-3-phosphate dehydrogenase (GAPDH) (60004-1-Ig; Proteintech, Rosemont, IL, USA), anti-F4/80 (ab6640; Abcam), microtubule-associated protein 2 antibody(anti-MAP2) (ab5392; Abcam), anti-CD16/32 (101325; Biolegend, San Diego, CA, USA), anti-Gr1-allophycocyanin (APC) (RM3005; eBioscience; San Diego, CA, USA), anti-CD11b-peridinin-chlorophyll-protein complex (PerCP) (45-0112-82; eBioscience), anti-F4/80-pohycoerythrin (PE) (MA5-16631; eBioscience), anti-CD45-fluorescein isothiocyanate (FITC) (11-0451-8; eBioscience), anti-β-III-tubulin (Covance, Princeton, NJ, USA) and neuronal nuclear antigen antibody (anti-NeuN) (Chemicon, Tokyo, Japan), LDH assay kit (A020-2-2; Nanjing Jiancheng Biology Engineering Institute, Nanjing, Jiangsu, China), and TUNEL assay kit (KeyGen Biotech, Nanjing, Jiangsu, China).

### Mouse model establishment and treatment

DBA/2J mice were purchased from Shanghai Jiesijie Laboratory Animals Co., Ltd. (Shanghai, China). The 4–6-week-old male and female mice were acclimated for 1 week before treatment. All experiments were approved by the Institutional Animal Research Committee and Ethics Committee of Zhejiang University (ZJU20170337). The typical diabetic model was established according to the method of Nguyen et al. [17]. In brief, pregnant mice (control model) were administered an intraperitoneal injection of streptozocin (STZ, 40 mg/kg) at 3 days, for 5 consecutive days. The level of fasting blood glucose (FBG) at 6 h after the last injection was not less than 11.1 mmol/L, successfully establishing a diabetic model. In our study, the chemerin-treated diabetic model was established by intraperitoneal injection of chemerin (3–4 μg/g body weight), according to the method of Yang et al. [18]. Then, the maternal diabetic was verified by measuring several indices, such as FBG and oral glucose tolerance test (OGTT).

According to chemerin-induced form of maternal diabetes, successfully mated female mice were randomly divided into three groups, controls, chemerin-treated mice, and chemerin treatment with ChemR23-knockdown/CCRL2-knockdown, which were constructed by an intravenous tail injection of 1 × 10^9^ plaque-forming units (pfu) ChemR23/CCRL2-short hairpin RNA (shRNA) lentivirus (Hangzhou Yingrui Science and Technology Co., Ltd., Hangzhou, China) on gestational day (GD) 10.5. The controls and diabetic mice were injected with 1 × 10^9^ pfu vehicle of lentivirus.

### Isolation of macrophages

Macrophages were isolated from 18.5-day-old fetal mice (E18.5; five to eight fetal brains) and peritoneal fluid of normal mice. Briefly, the offspring were killed with the brain exposed. The brain tissues were cut into pieces and filtered using a 200-mesh filter followed by washing with 5-ml phosphate-buffered saline (PBS). Then, the cell suspension was collected into a 15-ml centrifuge tube. After centrifugation at 1200 rpm for 5 min, the cell pellet was washed and resuspended in fluorescence-activated cell sorting (FACS) buffer. Then, the cells were stained with antibodies against CD45-FITC, CD11b-PerCP, and F4/80-PE and Gr-1-APC. CD^45high^CD11b^high^F4/80^high^, which represents the macrophage fraction, was sorted by a flow cytometer. The inner skin lining of the peritoneal cavity was exposed 3 days after an intraperitoneal injection of 3% thioglycollate, and 5 ml PBS (with 3% fetal calf serum) was injected into the peritoneal cavity to collect the macrophages. After gently massaging the peritoneum, the attached cells were dislodged into the PBS solution and as much fluid as possible was collected. The fluid was centrifuged at 1500 rpm for 8 min, and the cell pellet was resuspended and cultured in RPMI1640 medium for the following assay.

### FBG measurements

After fasting for 6 h, 200-μL venous blood was collected from the post-glomus venous plexus. The blood samples were centrifuged at 3000 rpm for 15 min at 4 °C, and the supernatant was used to detect the level of FBG by enzyme-linked immunosorbent assay (ELISA) according to the manufacturer’s instructions.

### Oral glucose tolerance test (OGTT)

After a 16-h fast, 2 g/kg glucose was given by gavage at GD18.5, and venous blood was collected at 0, 30, 60, 90, and 120 min after gavage, followed by the FBG measurement.

### ELISA

The ELISA kits for chemerin (tw039995), FBG (tw039025), IL-1β (tw040320), and IL-18 (tw040319), purchased from Shanghai Hongwei Co., Ltd. (Shanghai, China), were used to determine the levels in serum and supernatants. All assays were conducted strictly in accordance with the manufacturer’s instructions.

### Co-immunoprecipitation

The whole brain tissues from E18.5 (two fetal brains) were prepared for protein extraction using RIPA lysis buffer (P0013; Beyotime Biotechnology, Beijing, China), and total protein was quantified by the BCA protein assay kit (23225; Thermo). The protein solution was incubated with Protein A/G-Sepharose (20421; Thermo) and anti-chemerin overnight. After centrifugation and three washes in PBS, the polypeptides in the precipitated complexes were analyzed by western blotting.

### Western blotting

Total protein of one whole brain from E18.5 or B7 (7-day-old offspring) and peritoneal macrophages were extracted using the same method as for the co-immunoprecipitation assay. Protein samples were separated by 8–12% sodium dodecyl-sulfate polyacrylamide gel electrophoresis and transferred onto nitrocellulose membranes. The membranes were incubated with primary antibodies against chemerin (1:1000), ChemR23 (1:500), CCRL2 (1:1000), NLRP3 (1:2000), Asc (1:2000), caspase-1 (1:1000), caspase-3 (1:2000), caspase-7 (1:1000), caspase-8 (1:1000), IL-1β (1:500), IL-18 (1:500), and GAPDH (1:5000) for 2 h at room temperature or overnight, followed by exposure to horseradish peroxidase-conjugated anti-IgG secondary antibodies for 1.5 h. The membranes were incubated with an enhanced chemiluminescence buffer (32106; Thermo) and visualized with the gel documentation system (FluorChen E). The gray values of the targeted protein bands were detected using ImageJ 1.42q software (National Institutes of Health, Bethesda, MD, USA), and then, the ratio to GAPDH was calculated.

### FACS

Single-cell suspensions of the whole brain tissues from E18.5 (five to eight fetal brains) were purified by centrifugation and blocked using anti-CD/16/32 (1:200). Then, the cell suspension was treated with fluorescent antibodies against CD45-FITC (1:400), CD11b-PerCP (1:400), F4/80-PE (1:400), and Gr-1-APC (1:400)). Finally, these cells were sorted and counted by a flow cytometer (FACSCanto II; BD, Brea, CA, USA).

### Immunofluorescence staining

Immunocytochemistry was performed as described previously [19]. Briefly, a specimen of the tissue (forebrain, embryonic cortex, olfactory bulb, or dentate gyrus of adult offspring) was embedded in OCT compound, rapidly frozen in liquid nitrogen, and stored at − 80 °C. The embedded tissue was cut into 8–10 μm sections, which were fixed and rinsed in acetone and PBS, respectively. After blocking with goat serum, the sections were incubated with primary antibodies against chemerin, ChemR23, CCRL2, F4/80, MAP2, β-III-tubulin, and NeuN, followed by species-specific secondary antibodies. Macrophages from the brain tissues were sorted by FACS, seeded on coverslips, fixed in 4% paraformaldehyde, and permeabilized with 0.5% Triton X-100. The coverslips containing the cells were incubated with active caspase-1 antibody (ab1872; Abcam, Cambridge, UK) followed by species-specific secondary antibodies. Nuclei were counterstained with diaminobenzene. Last, the cells/sections were examined under a fluorescence microscope (Olympus, Tokyo, Japan), and the integrated optical density per unit area (IOD/Area) was evaluated using Image-Pro Plus 6.0 software (Media Cybernetics, Silver Spring, MD, USA).

#### Isolation, culture, and treatment of primary neurons

In brief, mice superior cervical ganglia (SCG) of E18.5 was cut and digested using mixed-digestive juice containing collagenase (2.5 mg/ml, Worthington), dispase (5 mg/ml, Roche Molecular Biochemicals), and trypsin (10 mg/ml, Worthington) for 20 min at 37 °C. After mechanical separation, non-neurons were separated out by extensive preplating. Then, the pure neurons were incubated in DMEM F12 medium supplemented with 3% FBS in 37 °C incubator with 5% CO_2_. In a 24-h incubation later, cells were conducted by TUNEL staining assay.

#### TUNEL staining

Coverslip pretreated with Poly-L-Lysine Solution was placed in a six-well plate and neurons were seeded at a density of 5 × 10^4^ cells/well. After incubation overnight, cells were exposed with PBS or 1, 5, and 10 nM chemerin for additional 24 h. Then, the slides were immersed 0.85% NaCl for 5 min at room temperature. Afterwards, sections were fixed using 10% formalin for 15 min and washed using PBS for twice. After that, slides were equilibrated for 5 min and incubated with 100 μL TdT reaction mix for 1 h at 37 °C in darkness. Stop solution was added to terminate the reaction followed by washing twice with PBS. After mounting using glycerol containing DAPI, images were observed by the fluorescence microscope.

### Quantitative real-time polymerase chain reaction (PCR)

The mRNA of one whole brain from E18.5 or 7-day-old offspring was extracted using the TaKaRa MiniBEST Universal RNA Extraction Kit. After purification, 1 μg mRNA was reverse-translated into cDNA with the PrimeScript® RT reagent Kit and gDNA Eraser (Takara), followed by real-time PCR using a PCR amplifier (ABI, Foster City, CA, USA). Relative quantitation was expressed as 2^−△△Ct^, where △Ct is the difference between the mean cycle threshold (Ct) value of duplicate measurements of the sample and GAPDH. The primer sequences were as follows: mouse NLRP3 forward 5′-ATGCTGCTTC-GACATCTCCT-3′ and reverse 5′-AACCAATGCGAGATCCTGAC-3′; mouse ASC forward 5′-GAAGCTGCTGACAGTGCAAC-3′ and reverse 5′-GCCACAGCTCC-AGACTCTTC-3′; mouse GAPDH forward 5′-AGGTCGGTGTGAAC-GGATTT-3′ and reverse 5′-TGTAGACCATGTAGTTGAGG-3′.

### Transwell assay

A 200-μL aliquot of the elicited macrophages (1 × 10^6^/mL) was transferred to the upper chambers, accompanied by 1, 10, 100, and 1000 nM chemerin/CXCL8 administration into the lower chambers. The Transwell chambers were taken out and washed with calcium-free PBS 24 h later and then fixed with 4% methanol. After removing the non-migrating cells from the upper layer, the migrated cells were stained with 0.1% crystal violet and examined under a microscope.

### Cell death assay

Pyroptotic cell death was evaluated with LDH release assay. For LDH release, cell culture supernatants were collected and the LDH activity was detected using the LDH assay kit (Nanjing Jiancheng Biology Engineering Institute, Nanjing, Jiangsu, China). Briefly, 25-μL cell supernatant and 25 μL substrate were mixed together and incubated at 37 °C for 15 min. Then, 25-μL 2,4-dinitrophenylhydrazine was added into the samples and incubated at 37 °C for 15 min. Finally, 250-μL 0.4 mol/L NaOH solution was added and incubated at room temperature for 5 min. The absorbance was measured at 450 nm on a spectrophotometric microplate reader.

### Behavioral assessments

The open field test (OFT) used a homemade open field (OF) box with a black wall and bottom (80 × 80 × 40 cm). The bottom of the OF apparatus was divided into 25 equal-area squares marked by white lines. Mice were put in the OF apparatus 1 h before the OFT to allow them to gradually acclimatize to their surroundings. At 8 weeks of age, offspring in each group were placed in the center of the apparatus and exploratory activity, including horizontal activity (crossing frequency between squares and frequency of crossing the center squares) and vertical activity (rearing frequency and rearing time), and were recorded using a video camera and analyzed by computer software.

### Statistical analysis

All experiments were performed with at least three replicates. The statistical analyses were performed using IBM SPSS software (ver. 22.0; IBM Corp., Armonk, NY, USA). Group means were compared using the two-sided Student’s *t* test (Bonferroni correction was performed when necessary). All data are presented as mean with 95% confidence interval (95% CI). A *P* value < 0.05 was considered significant.

## Results

### Maternal chemerin injections in pregnancy successfully induce a form of maternal diabetes

**Fig. 1:**
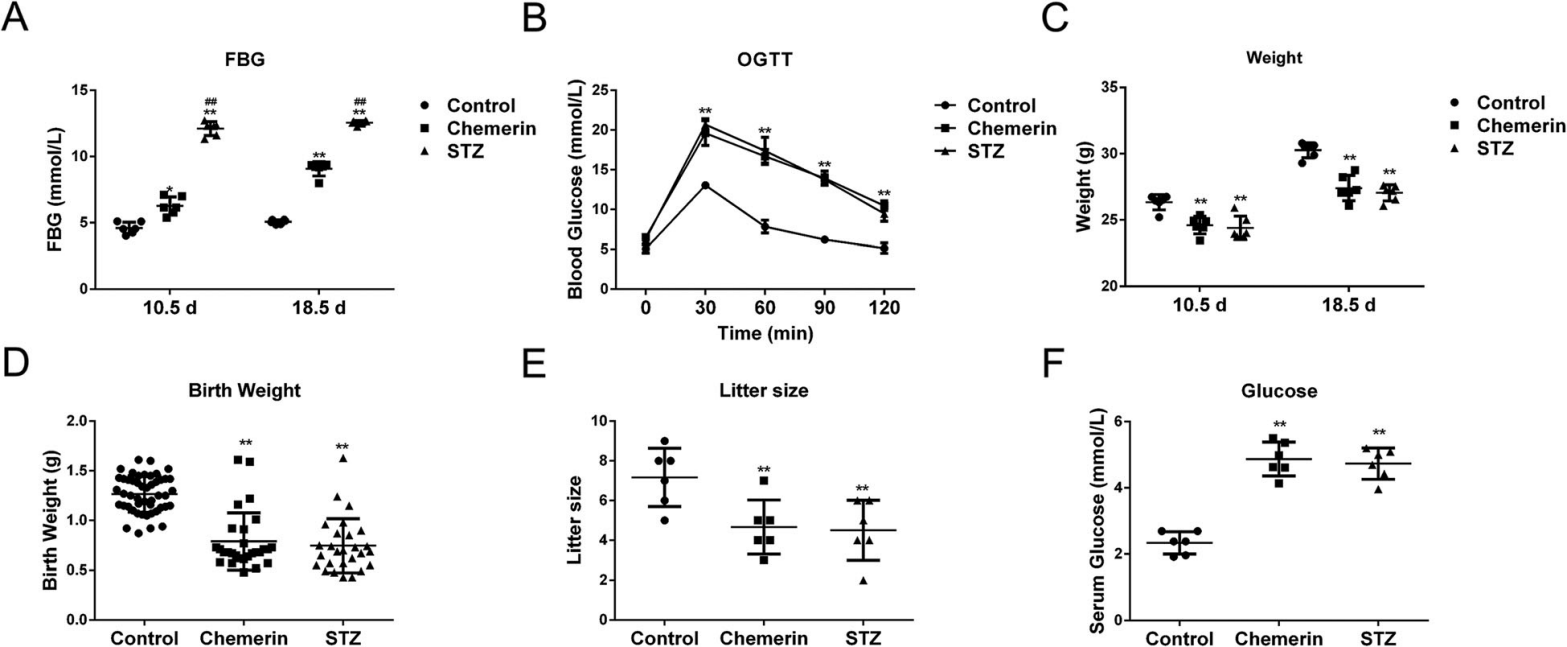
Chemerin-induced diabetic pregnant model. **a** Measurements of fasting blood glucose (FBG) in the advanced stages of pregnancy (GD10.5 and GD18.5) by enzyme-linked immunosorbent assay (ELISA). **b** The oral glucose tolerance test (OGTT) results at GD18.5. **c** Body weight of mice at GD10.5 and GD18.5. **d** Mean birth weight of control, streptozocin (STZ), and chemerin-treated mice. **e** Analysis of litter size of the control, STZ-treated dams, and the chemerin injected groups. **f** The FBG level in 28-day-old offspring. Data are mean with 95% confidence interval (CI). *n* = 6 for all groups. *P < 0.05, **P < 0.01 vs. controls in the same time; #*P* < 0.05, ##*P* < 0.01 when STZ induced diabetes dams vs. chemerin induced diabetes dams in the same time

Chemerin is involved in metabolic diseases; however, the mechanism is still unclear. To verify the association between chemerin and the pathology of maternal diabetes and its associated complications, we first established the maternal diabetes mice model using a new method, defined as chemerin-induced diabetic mouse dams in this study. The STZ-mediated diabetic pregnant mice were the most commonly used in previous studies. Thus, detection of indicators in STZ-induced diabetic dams was used as the positive reference value to evaluate the symptom of maternal diabetes model established in this study. The levels of FBG increased significantly in the chemerin and STZ treatment groups compared to controls. Higher levels of FBG occurred at GD10.5 and GD18.5 in mice treated with STZ than in those treated with chemerin (Fig. 1a). The levels of blood glucose on the OGTT test peaked at 30 min and then declined gradually after peaks at 60, 90, and 120 min, in all groups. However, the blood glucose levels of mice injected with STZ or chemerin increased 1.5-fold compared to the control mice from 30 to 120 min, suggesting that STZ-treated and chemerin-treated mice had developed serious glucose intolerance (Fig. 1b). However, both chemerin- and STZ-treated groups lost body weight at GD10.5 and GD18.5, and there was no difference in body weight between the chemerin- and STZ-treated groups (Fig. 1c). The average neonatal birth weight per litter and litter size decreased significantly in mice injected with chemerin or STZ compared to the control mice (Fig. 1d, e). Though the average neonatal birth weight was declined, both chemerin and STZ treatment had macrosomia: 2 macrosomia in chemerin-treated group (28 offspring) and 1 macrosomia in STZ-treated group (27 offspring). Additionally, the FBG level at the age of 28 days increased in the offspring of mice injected with chemerin or STZ relative to the controls (Fig. 1f). These results indicate that chemerin injection mimicked the characteristics of maternal diabetes which is in consistent with the phenotype of STZ-induced diabetic pregnant dams, and thus was suitable for subsequent experiments.

### Chemerin-mediated maternal diabetes causes a decrease in the number of neurons and impaired recognition memory in offspring

**Fig. 2:**
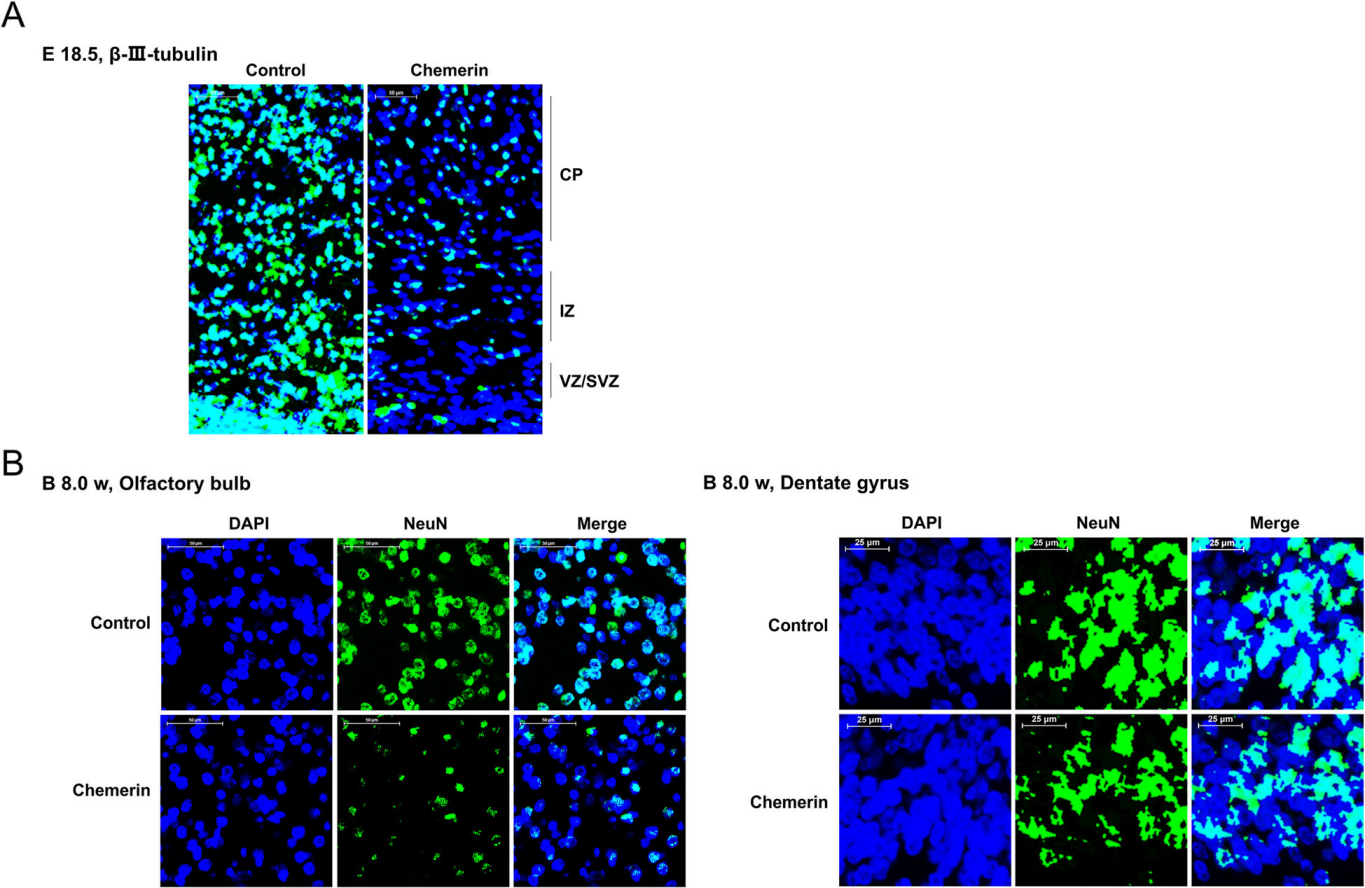
Effects of chemerin on neuronal development in the embryonic cortex and in 2-month-old offspring. **a** The expression and distribution of β-III-tubulin in coronal cortical sections at E18.5 as analyzed by immunofluorescent staining. CP, cortical plate; IZ, intermediate zone; VZ/SVZ, ventricular and subventricular precursor zones. DAPI: blue; β-III-tubulin: green. Scale bar: 50 μm. **b** Olfactory bulb (scale bar, 50 μm) and dentate gyrus (scale bar, 25 μm) of 8-week-old offspring were conducted for immunofluorescent staining with antibody against NeuN. DAPI: blue; NeuN: Green

Diabetes-related inflammation in the mother during embryogenesis perturbs brain development in the offspring [20, 21]. We next explore the impacts of chemerin administration on neurodevelopment and behavioral features of offspring from chemerin-induced diabetic dams. As shown in Fig. 2a, sections of E18.5 cortex were immunostained with β-III-tubulin, which is an early biomarker of neural cell differentiation, to analyze the role of chemerin in the development of the embryonic murine cortex. The results showed that the total number of β-III-tubulin-positive cells decreased sharply in the chemerin-induced group compared to the control group, in the intermediate zone (IZ), the cortical plate (CP), and the ventricular and subventricular precursor zones (VZ/SVZ), which are regions containing newborn neurons (Fig. 2a and Additional file 1: Figure S1A). Besides, neuronal nuclear antigen NeuN, a biomarker of mature neurons, also decreased significantly in the olfactory bulb and dentate gyrus under the chemerin treatment condition, indicating fewer neurons in the offspring (8 weeks old) of the chemerin-treated group compared to the control mice (Fig. 2b and Additional file 1: Figure S1A). These data suggested that chemerin administration in pregnancy causes a decline in the number of neurons in offspring.

**Fig. 3:**
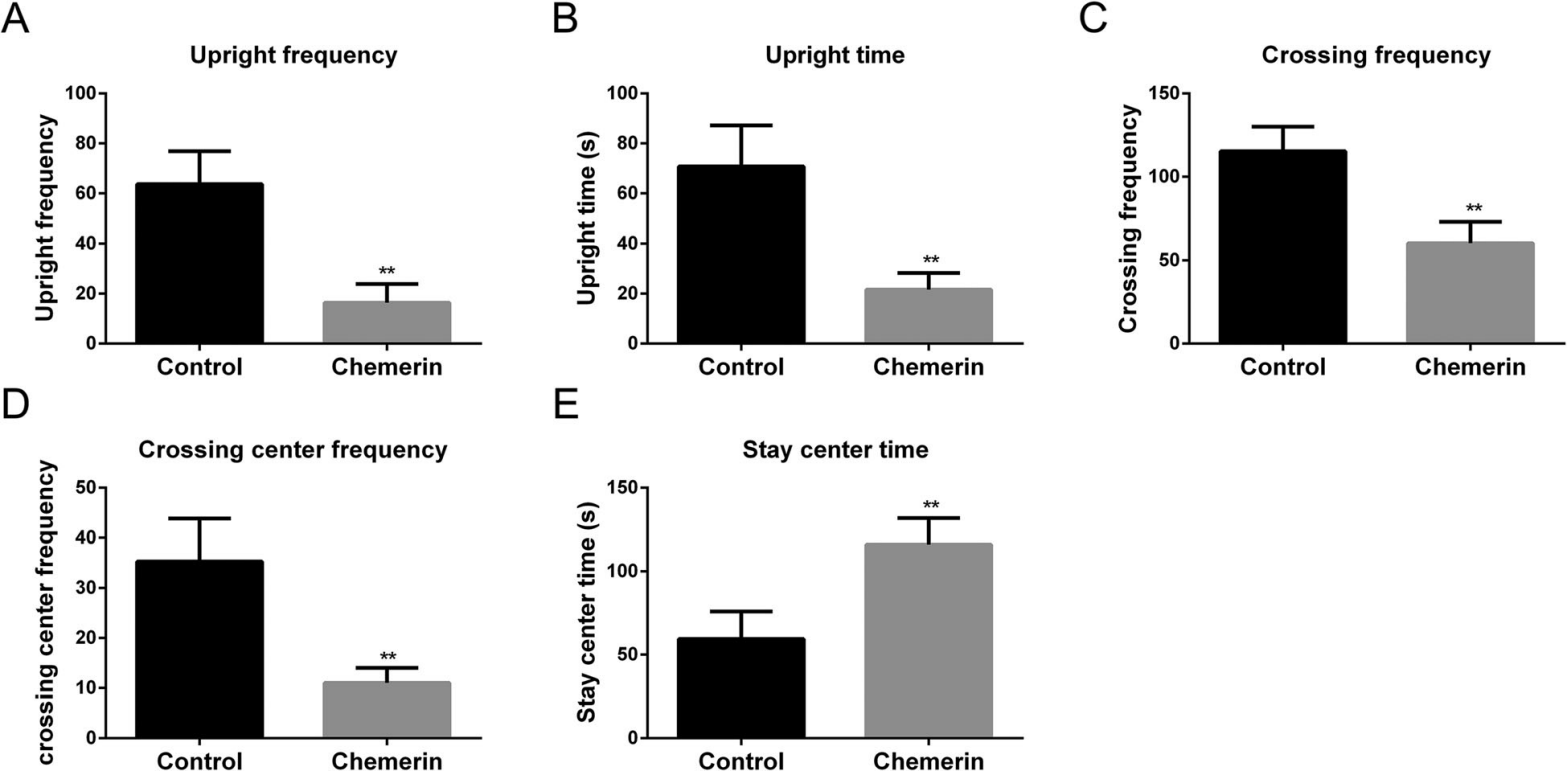
Recognition memory of the offspring of diabetic dams. Rearing frequency (**a**) and rearing times (**b**) of 8-week-old offspring from a normal pregnancy and from chemerin-mediated diabetic dams. Examination of crossing frequency between squares (**c**) and frequency of crossing of the center squares (**d**) by 8-week-old offspring. (**e**) Immobility time in 8-week-old offspring. *Chemerin-induced diabetic group vs. controls. **P < 0.01

Disturbed neuronal circuitry could impair cognitive ability. We further analyzed the role of chemerin in offspring behavior by analyzing differences in horizontal and vertical activities between offspring from the control and chemerin-induced mice groups using the OFT. Shorter rearing time and lower rearing frequency were observed in the offspring from chemerin-treated group (8 weeks old) compared to the control group (Fig. 3a, b). Horizontal activities, including the crossing frequency between squares and the frequency of crossing the center squares, decreased in the offspring of diabetic mice (Fig. 3c, d), while immobility time (staying in the center) was clearly prolonged (Fig. 3e).

These data suggest that the neural development and behavior of offspring from chemerin-induced diabetic mice were impaired, implying that the brain of offspring from diabetic dams may be injured or inflamed.

### Chemerin is recruited into the brain tissues of offspring from mice with diabetic dams dependent on CCRL2

To explore why offspring from chemerin-induced diabetic mice presented with a decrease in the number of neurons and impaired recognition memory, we focused on the pathological changes in brain tissues of diabetic dams’ offspring, particularly on the chemerin-related changes. Based on the chemerin-induced maternal diabetes model, we first analyzed the levels of chemerin in brain tissues of dams' fetuses and their offspring. As shown in Additional file 1: Figure S1, the chemerin protein level was robustly enhanced in brain tissues of 18.5-day-old fetal mice and 7-day-old offspring from chemerin-exposed mice compared to controls, suggesting that chemerin might be enriched in the offspring’s brain (Additional file 1: Figure S1B).

**Fig. 4:**
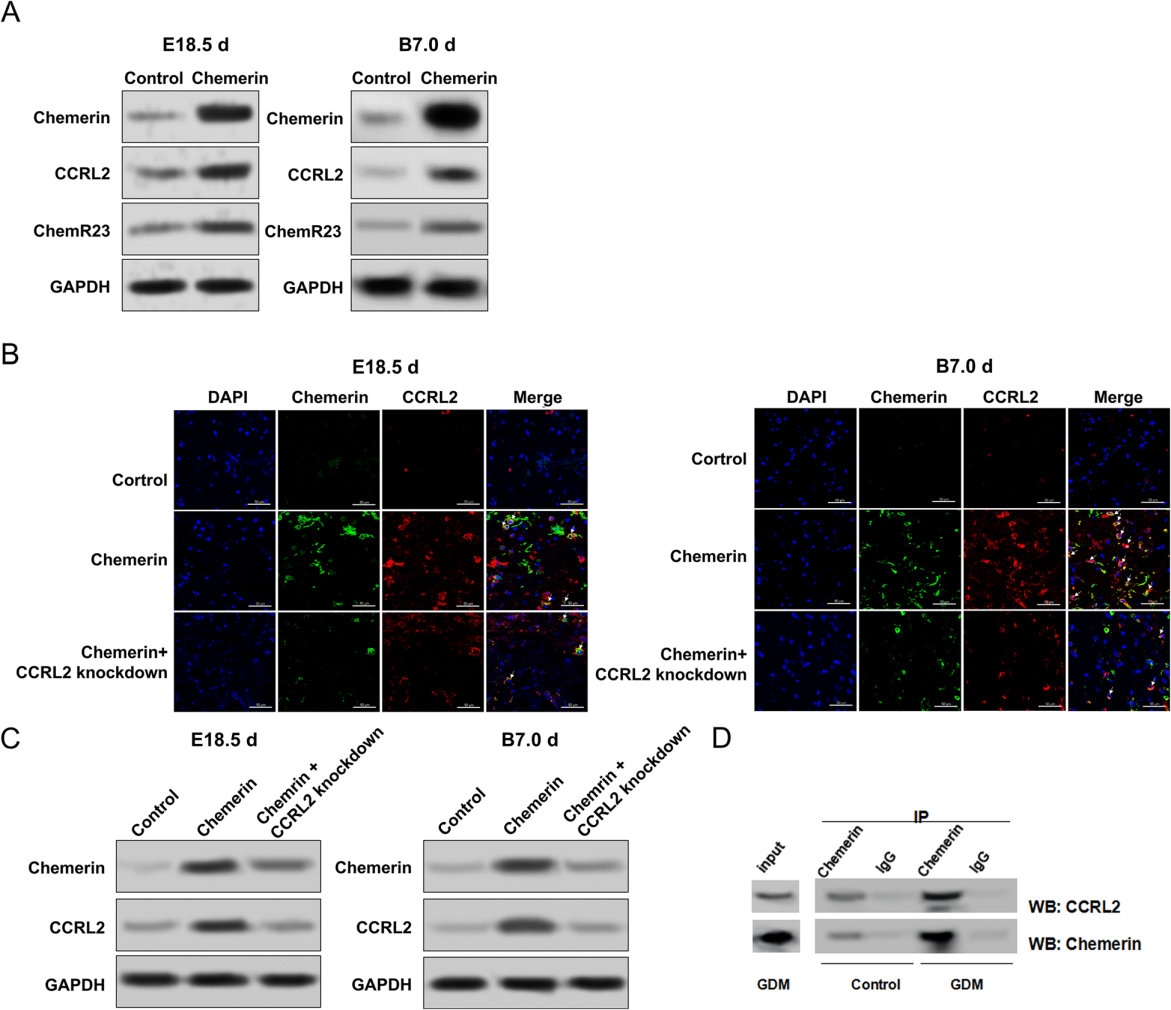
Association between chemerin enrichment and chemokine (C-C motif) receptor-like 2 (CCRL2) in 18.5-day-old fetal mice and 7-day-old offspring. **a** Protein levels of chemerin, CCRL2, and ChemR23 in whole brain tissues of 18.5-day-old fetal mice and 7-day-old offspring from controls and chemerin-induced diabetic mice (tissues from one whole brain). **b** Immunofluorescence staining for chemerin and CCRL2 in forebrain tissue specimens of offspring from the control, chemerin-induced diabetic dams, and chemerin-induced diabetic dams with CCRL2-knockdown mice. **c** Detection of chemerin and CCRL2 protein expression in brain tissue by western blotting (tissues from one whole brain). **d** The interaction between chemerin and CCRL2 was evaluated by co-immunoprecipitation and immunoblot assays in brain tissues of 18.5-day-old fetal mice (two fetal brains). Scale bar: 50 μm

Chemerin interacts with its receptors. Thus, we also assessed the levels of CCRL2 and ChemR23, which are chemerin receptors activated during chemerin-mediated signaling [22]. Interestingly, both CCRL2 and ChemR23 were enhanced in the brain tissues of 18.5-day-old fetal mice and 7-day-old offspring from the chemerin-induced maternal diabetes group (Fig. 4a). It has been reported that CCRL2, an atypical chemerin receptor highly expressed in brain cells, increases the local concentration of chemerin and presents chemerin to leukocytes expressing ChemR23 [22–24]. Therefore, aggregation of CCRL2 possibly occurs in response to the increase of chemerin via a feedback mechanism.

Previous studies have suggested that CCRL2 plays a leading role in chemerin enrichment, and we speculated that the increase in CCRL2 might have selective signaling properties in chemerin-mediated diabetic mice. Therefore, an additional group of CCRL2-knockdown mice was used to evaluate why chemerin accumulated progressively in the brain tissues of offspring from chemerin-treated mice. The blood-embryo barrier (BEB) prevents ectogenic macromolecules, such as chemerin, from entering fetal circulation. However, maternal macromolecules could possibly enter fetal circulation when the BEB is impaired [25]. An aberrant anatomical structure, such as injured intercellular tight junctions, has been observed in the placenta of diabetic pregnant patients [26]. Thus, an intravenous tail injection of CCRL2 or other gene-shRNA lentivirus could enter the fetal circulation through an injured BEB. In fact, CCRL2 in fetal mice and offspring from chemerin-evoked dams was downregulated after an injection of CCRL2-shRNA, and the knockdown efficiency is illustrated in Additional file 2: Figure S2A. First, immunofluorescence results for the forebrain tissue of 18.5-day-old fetal mice or 7-day-old offspring from the chemerin-launched model indicated that chemerin (green) was significantly enriched and accompanied by enhancement of CCRL2 (red), while the accumulation of chemerin was clearly suppressed in chemerin-treated mice with CCRL2-knockdown (Fig. 4b). The IOD of chemerin- and CCRL2-positive cells was measured with computerized image processing, and the results confirmed that CCRL2-knockdown prevented chemerin from accumulating in the offspring brain (Additional file 2: Figure S2B). The accumulation of chemerin in chemerin-treated mice decreased during CCRL2 depletion, even though there was still more chemerin than in the control (Fig. 4c). A co-immunoprecipitation assay was conducted to identify the role of CCRL2 in the process of chemerin enrichment, and we observed that the interaction between chemerin and CCRL2 increased in 18.5-day-old fetal mice brain tissues from diabetic dams (Fig. 4d). Besides, the measured level of chemerin in the brain tissue of E18.5 and 7-day-old offspring was significantly decreased in the absence of CCRL-2 (Additional file 1: Figure S1B). Therefore, the enrichment of chemerin in the offspring brain partly depends on the presence of CCRL2 in the brain tissue of the offspring.

### Macrophages are recruited by enriched chemerin in the brains of offspring of chemerin-induced diabetic dams

**Fig. 5:**
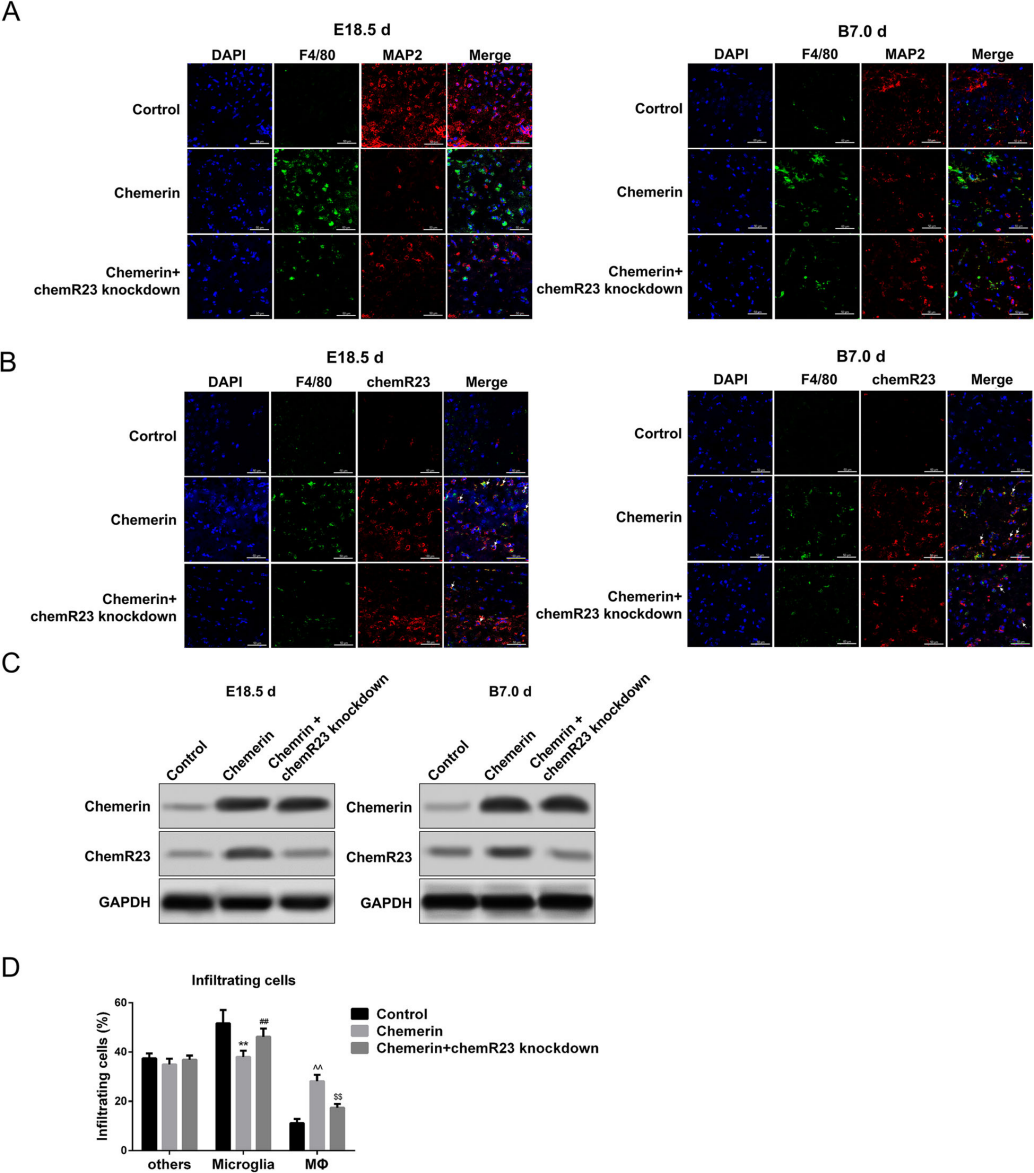
Macrophage recruitment by chemerin in the brain tissue. Immunofluorescence staining for F4/80 (macrophages) and MAP2 (neurons) (**a**) and ChemR23 and F4/80 (**b**) in the forebrain tissue of 18.5-day-old fetal mice and 7-day-old offspring from control, chemerin-induced diabetic dams, and chemerin-induced diabetic dams with ChemR23 knockdown mice. **c** Chemerin and ChemR23 protein levels in the brain tissues of 18.5-day-old fetal mice and 7-day-old offspring from controls and chemerin-induced diabetic dams (tissues from one whole brain). **d** Infiltrating cell rates in brain tissues of 18.5-day-old fetal mice. Macrophages, microglia, and other cell fractions were sorted by fluorescence-activated cell sorting (FACS) (five to eight fetal brains). Data are mean with 95% CI. *Microglia from chemerin-induced diabetic group vs. microglia from controls; #Microglia from chemerin-induced diabetic group with ChemR23 knockdown vs. microglia from chemerin-induced diabetic group. ^Macrophage from chemerin-induced diabetic group vs. macrophage from controls; $Macrophage from chemerin-induced diabetic group with ChemR23 knockdown vs. macrophage from chemerin-induced diabetic group. **, ##, ^^ and $$—*P <* 0.01. Scale bar: 50 μm

The mechanism by which the accumulation of chemerin in the brain tissue of the offspring results in a decrease in neurons and aberrant behavior remains unclear. Some studies indicate that the inflammation-modulating effect of chemerin relies on ChemR23, which is mainly expressed in macrophages and dendritic cells in brain tissues [15]. Therefore, we also assessed macrophage infiltration in brain tissues. Immunofluorescence staining revealed that the numbers and IOD of macrophages (green, F4/80-positive cells) were upregulated in the forebrain tissue of 18.5-day-old fetal mice and 7-day-old offspring from chemerin-treated mice compared to controls; coincidently, the numbers and IOD of nerve cells (red, MAP2-positive cells) were simultaneously downregulated in these offspring forebrain tissues (Fig. 5a and Additional file 2: Figure S2C). Importantly, the enrichment of macrophages (marked with F4/80, green) was accompanied by downregulation of neurons co-located with the accumulation of chemerin in forebrain tissue of 18.5-day-old fetal mice and 7-day-old offspring (Fig. 5b and Additional file 2: Figure S2D). These data confirm that chemerin recruitment indeed mediated the migration of macrophages to the site of inflammation in offspring brain tissue, which might be associated with the decrease in neurons.

Based on these results, we used ChemR23-knockdown mice to further evaluate the role of ChemR23 in chemerin-macrophage-neuron changes. As shown in Fig. 5a and b, ChemR23-knockdown robustly reduced chemerin-mediated enhancement of macrophages (green) and restored the MAP2-positive cells (red) in the forebrain tissue of 18.5-day-old fetal mice and 7-day-old offspring from chemerin-treated mice relative to control mice (Fig. 5a, b and Additional file 2: Figure S2C-S2D). ChemR23-shRNA lentivirus also crossed the blood-brain barrier (BBB) and entered the fetal circulation because of the knockdown efficiency of ChemR23 (Additional file 2: Figure S2A). Chemerin expression in the offspring of diabetic dams was not different whether ChemR23 was knocked down or not, indicating that ChemR23 had no effect on the enrichment of chemerin in the brain tissue of offspring from diabetic mice (Fig. 5b, c and Additional file 2: Figure S2D). Utilizing FACS, we further explored the regulatory role of chemerin and ChemR23 on macrophage aggregation. As shown in Additional file 3: Figure S3A, the CD45^intermediate^CD11b^intermediate^ population represented the microglial fraction, and CD^45high^CD11b^high^F4/80^high^ represented the macrophage fraction. FACS demonstrated enhancement of the proportion of infiltrating inflammatory cells (macrophages) and a decrease in microglial cells, in the chemerin treatment group, but removing ChemR23 partly restored the microglial cells and inhibited the accumulation of macrophages (Fig. 5d).

In the in vitro experiment, the expression of ChemR23 in macrophages isolated from the peritoneal fluid of normal mice increased when stimulated by 10–1000 nM chemerin; the greatest effect was observed at 10 nM (Additional file 3: Figure S3B). Chemotactic migration of macrophages towards the chemerin accumulation site was observed at the optimum concentration of 10 nM in the Transwell assay (Additional file 3: Figure S3C). These results demonstrate that chemerin enrichment contributes to chemotactic migration of macrophages towards the brain tissues of offspring of diabetic mice. Chemerin promotes the increase in ChemR23, which may be mediated by the accumulation of macrophages and/or a direct modulatory effect.

To exclude the direct toxicity of chemerin which was recruited in offspring’s brain by CCRL2 on nerve cells, we firstly evaluated the expression distribution of ChemR23 in the forebrain tissue of E18.5 and 7-day-old offspring from diabetic dams. Through the IF staining assay, accompanied by the upregulation of ChmR23, we observed that chemerin administration also induced the accumulation of macrophages (green, F4/80) and the decline of neurons (gray, MAP2) in the brain tissue of E18.5, whose alterations were more noticeable in offspring’s brain from diabetic dams (7 days old). Importantly, ChemR23 was expressed most heavily in the macrophages, but very little in the neurons (Additional file 4: Figure S4A). Furthermore, the direct role of chemerin on primary neurons was conducted. After the conversion, the concentration of chemerin which crossed the placenta to the fetal brain was 6.25 nM (Additional file 1: Figure S1B). When exposed with 1, 5, and 10 nM chemerin, the number of apoptotic neurons was unchanged compared to control cells exposed with PBS (Additional file 4: Figure S4B). Collectively, these data confirm that chemerin-mediated decrease of neurons was indirectly through the recruitment of inflammatory cells, but not through the direct toxicity to the fetal brain.

### Chemerin induces the formation of the NLRP3 inflammasome and activates pyroptosis in macrophages

**Fig. 6:**
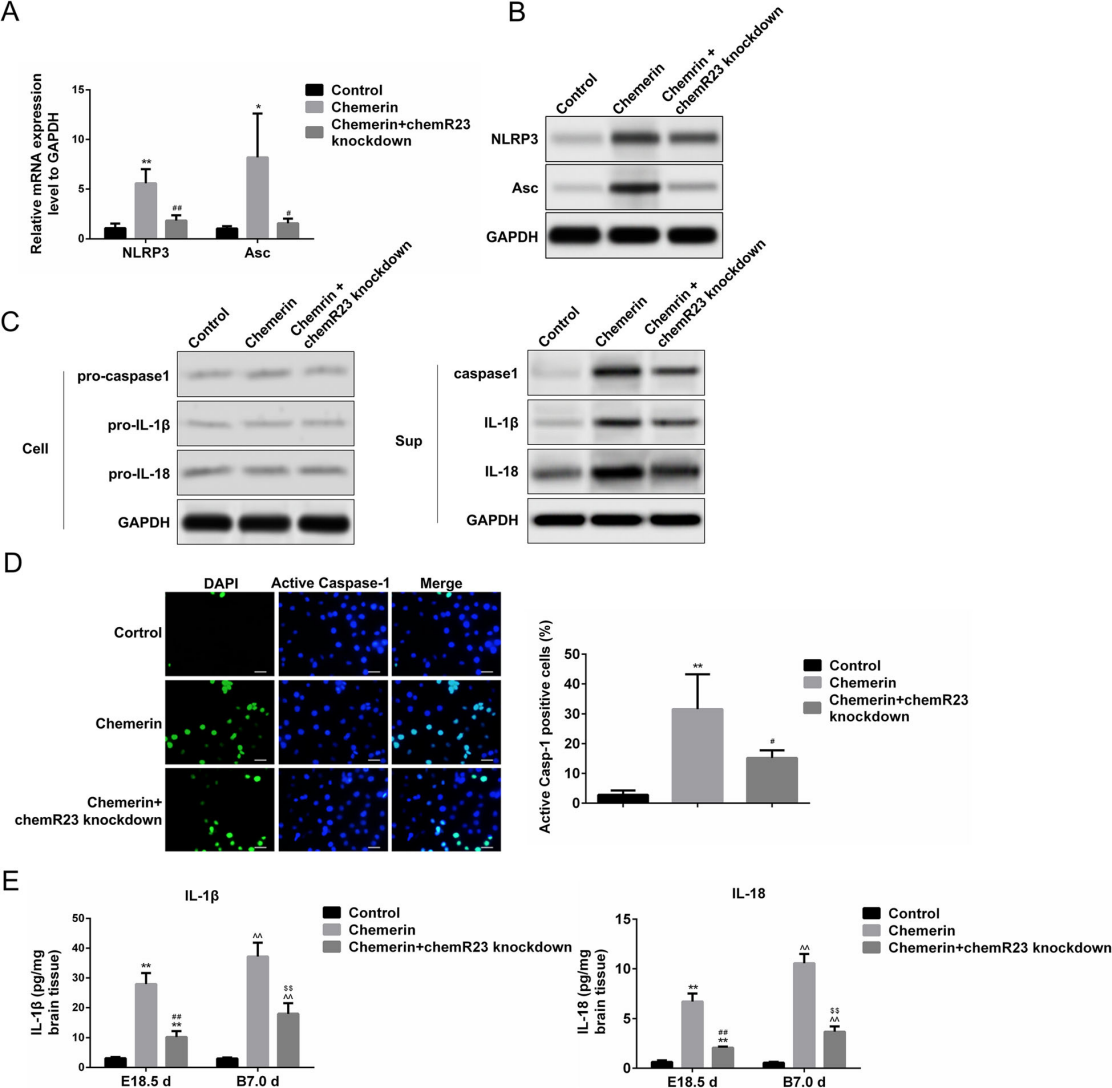
Activation of pyroptosis in fetal mice and offspring of diabetic dams. mRNA (**a**) and protein (**b**) levels of NOD-like receptor family pyrin domain containing 3 (NLRP3) and apoptosis-associated speck-like protein (Asc) in macrophages isolated from brain tissue of 18.5-day-old fetal mice (five to eight fetal brains). **c** Protein levels of pro-caspase-1, pro-interleukin (IL)-1β, and pro-IL-18 in macrophages (left) and levels of caspase-1, IL-1β, and IL-18 in the culture supernatants of macrophages (right). Glyceraldehyde-3-phosphate dehydrogenase (GAPDH) served as the internal control. **d** Active caspase-1-positive cells in macrophages isolated from the brain tissues of 18.5-day-old fetal mice. The levels of IL-1βand IL-18 (**e**) in the brains of 18.5-day-old fetal mice and 7-day-old offspring (pools of three whole brains). *Chemerin-induced diabetic dams vs. controls (E18.5 days), #chemerin-induced diabetic dams with ChemR23 knockdown vs. chemerin-induced diabetic dams (E18.5 days), ^chemerin-induced diabetic dams vs. controls (B7.0 days), and $chemerin-induced diabetic dams with ChemR23 knockdown vs. chemerin-induced diabetic dams (B7.0 days). * and #—*P <* 0.05; **, ##, ^^, and $$—*P <* 0.01. Scale bar: 50 μm

To further assess the role of chemerin-recruited macrophages in the pathological changes in offspring brains, we determined the NLRP3 inflammasome level in macrophages, which is associated with inflammation during chemerin treatment [16]. Quantitative real-time PCR and western blotting revealed that the level of the NLRP3 inflammasome and apoptosis-associated speck-like protein (Asc) were clearly promoted in macrophages isolated from the brain tissue of 18.5-day-old fetal mice (chemerin-induced diabetic mice group). However, ChemR23-knockdown inhibited chemerin-mediated enhancement of NLRP3 and Asc expression (Fig. 6a, b). The NLRP3 inflammasome mediates pyroptosis, which is characterized by activation of caspase-1 and secretion of pro-inflammatory cytokines, such as IL-1β and IL-18, mainly by infiltrating macrophages [27, 28]. We detected the level of pyroptosis-associated protein during cell lysis, and in the culture supernatants of the abovementioned macrophages, in Fig. 6. The levels of cleaved caspase-1, IL-1β, and IL-18 increased significantly in macrophages of offspring from the chemerin-induced diabetic dams group, in which expressions were notably inhibited in the ChemR23-knockdown group (Fig. 6c, right panel). However, no differences were observed in the expression of the precursors of these proteins (pro-caspase-1, pro-IL-1β, and pro-IL-18) among the groups (Fig. 6c, left panel). These results prompted us to explore the pyroptosis pathway in macrophages rather than the apoptotic pathway. Similarly, active caspase-1-positive cells were significantly more frequent in the macrophages isolated from fetal mice (E18.5) from diabetic mice than those from the control group, but the increase was suppressed in the macrophages isolated from the offspring of chemerin-evoked diabetic dams with ChemR23-knockdown (Fig. 6d). High levels of IL-1β and IL-18 were detected in the brain tissue of 18.5-day-old fetal mice and 7-day-old offspring from mice in the chemerin-treated group compared to the control group, which were rescued by ChemR23 depletion (Fig. 6e).

**Fig. 7:**
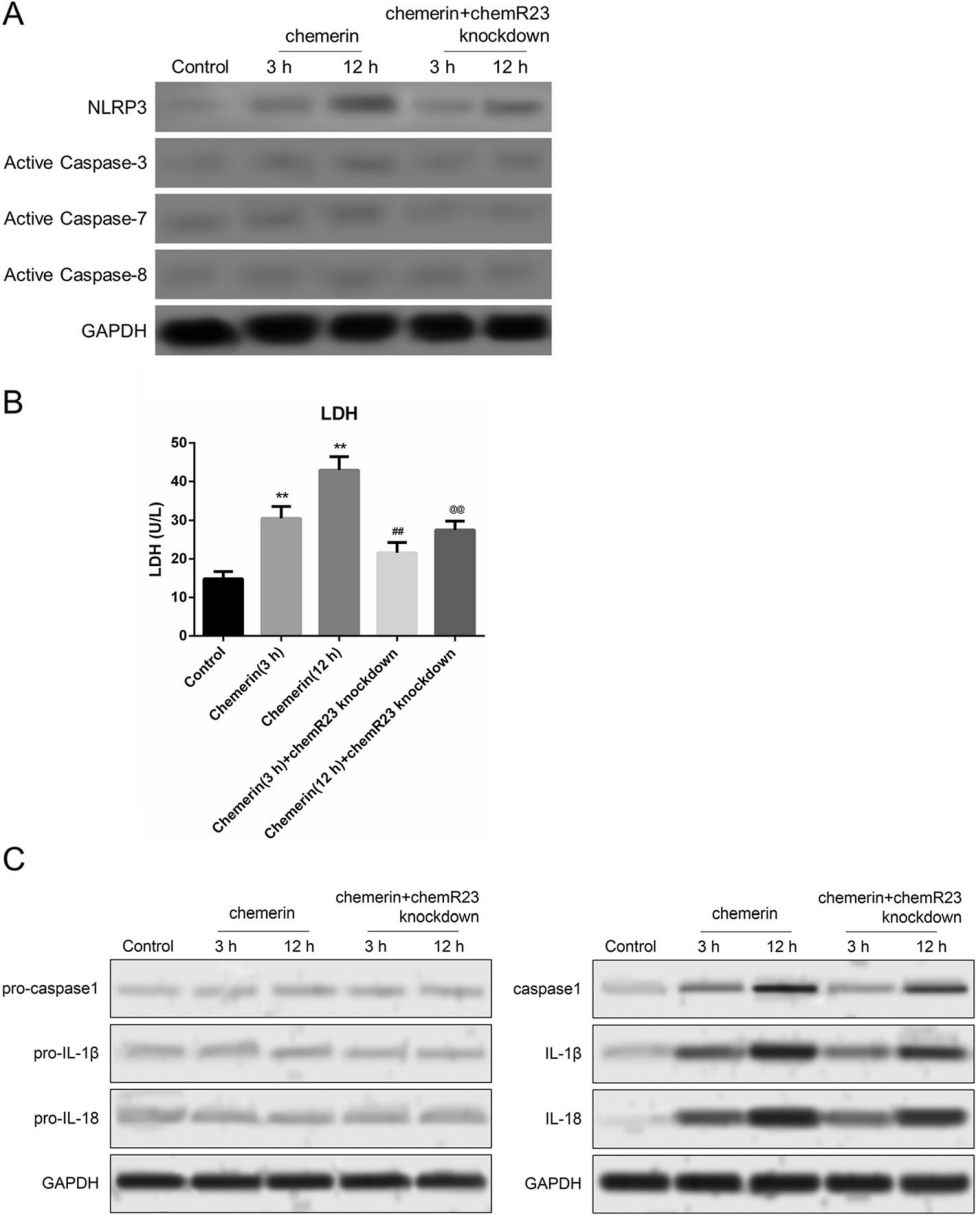
Role of chemerin in the activation of pyroptosis in vitro. **a** Protein levels of NLRP3, active caspase-3, active caspase-7, and active caspase-8 were detected by western blotting in macrophages treated with chemerin. Macrophages were isolated from the peritoneal cavity of normal mice and stimulated with 10 nM chemerin for 3 and 12 h, respectively. **b** Measurement of lactate dehydrogenase (LDH) activity in macrophages treated with chemerin for 3 and 12 h. **c** Protein expression of pro-caspase-1, pro-IL-1β, and pro-IL-18 in macrophages (left) and the levels of caspase-1, IL-1β, and IL-18 in the culture supernatants of macrophages (right) under the chemerin treatment condition. *Chemerin group vs. controls. #Chemerin group with ChemR23 knockdown vs. chemerin group, under the chemerin treatment for 3 h. @Chemerin group with ChemR23 knockdown vs. chemerin group, under chemerin treatment for 12 h. **, ##, and @@—*P <* 0.01

We also verified chemerin-induced activation of pyroptosis in macrophages isolated from the peritoneal cavity of mice in vitro. The NLRP3 inflammasome was induced by stimulation with chemerin for 3 and 12 h and was highly expressed at 12 h. On the other hand, removing ChemR23 blocked the chemerin-mediated increase in NLRP3 expression **(**Fig. 7a). The knockdown efficiency of ChemR23 in macrophages is illustrated in Additional file 2: Figure S2A. However, chemerin treatment did not induce the expression of active caspase-3, active caspase-7, or active caspase-8, indicating that chemerin-mediated brain injury is not regulated by the progression of cell apoptosis (Fig. 7a). Similar to NLRP3, the activity of lactate dehydrogenase (LDH) was promoted in macrophages during the chemerin treatment and partly attenuated in the absence of ChemR23 (Fig. 7b). Additionally, we observed no changes in the precursors of caspase-1, IL-1β, or IL-18 during cell lysis of macrophages. However, in the culture supernatants of macrophages, the release of caspase-1, IL-1β, and IL-18 increased tremendously in response to chemerin for 3 and 12 h and this promoting effect was impaired in macrophages treated with chemerin and ChemR23-knockdown (Fig. 7c). These data indicate that chemerin mediates pyroptosis of macrophages in brain tissues, possibly by interacting with ChemR23.

### ChemR23 and CCRL2 depletion ameliorate the inhibition of neural development and impaired recognition memory

**Fig. 8:**
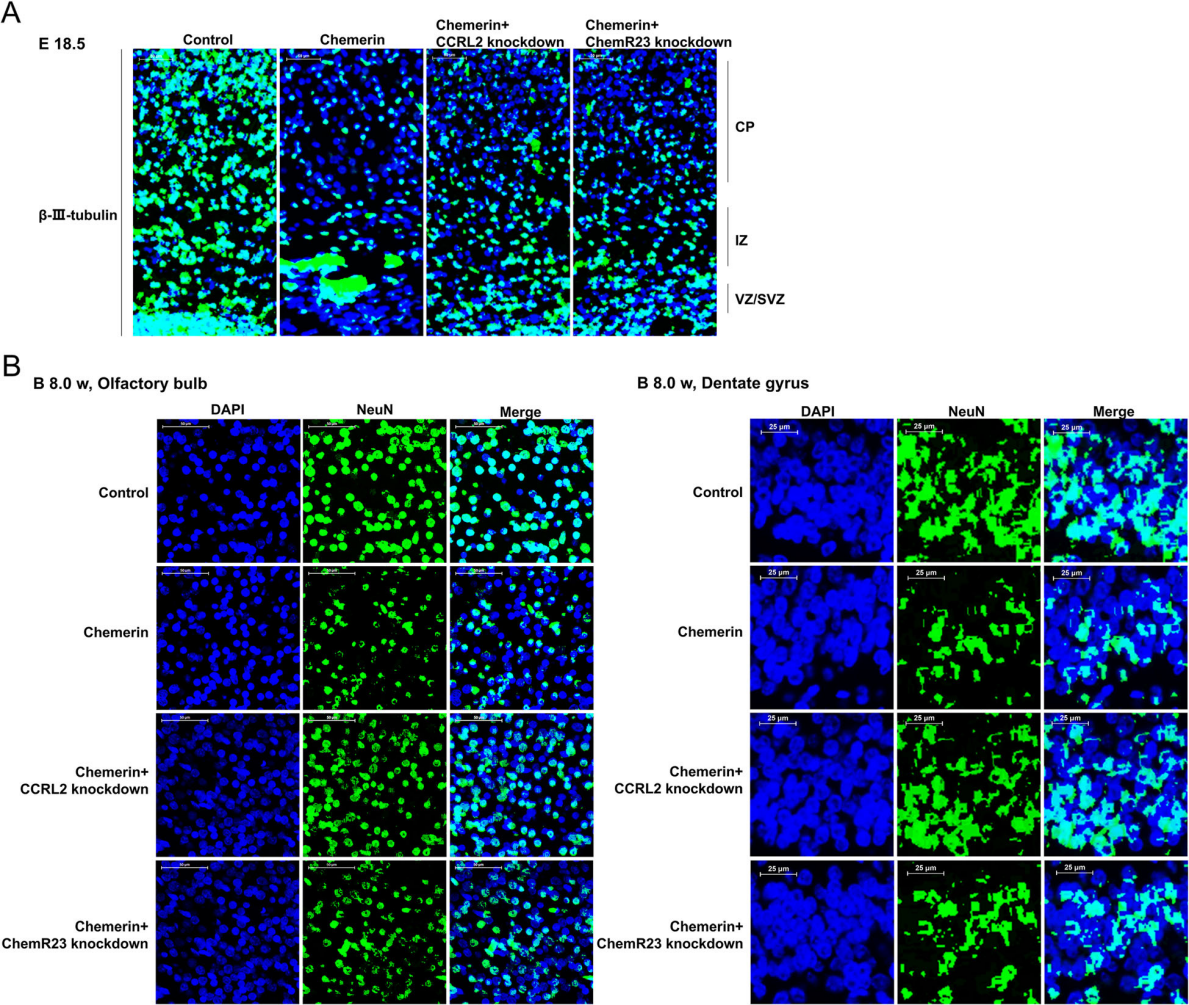
Effects of CCRL2 and ChemR23 on neuronal development in the embryonic cortex and in 8-week-old offspring. **a** Coronal cortical sections at E18.5 were analyzed by β-III-tubulin-immunofluorescent staining. DAPI: blue; β-III-tubulin: green. Scale bar: 50 μm. **b** Olfactory bulb (scale bar, 50 μm) and dentate gyrus (scale bar, 25 μm) of 8-week-old offspring were processed for immunofluorescent staining with NeuN antibody. DAPI: blue; NeuN: green

Given that chemerin treatment and activation of NLRP3 triggers the inflammatory response and pyroptosis, leading to neurological damage [29, 30], we speculate that removing ChemR23 and CCRL2 may relieve chemerin-mediated neuron loss and cognitive impairment by inhibiting the formation of the NLRP3 inflammasome. To verify the important roles of ChemR23 and CCRL2 with respect to our hypothesis, and to explore the potential therapeutic value of these targets, we knocked down ChemR23 and CCRL2 and observed the changes in neurodevelopment and behavioral features of offspring from chemerin-induced diabetic dams. As shown in Fig. 8a, the results showed that the total number of β-III-tubulin-positive cells were robustly decreased in the IZ and CP in the chemerin-induced group compared to the controls, and moderate aggregation was seen in the VZ/SVZ. The distribution and the total number of β-III-tubulin-positive cells notably increased in the VZ/SVZ, IZ, and CP regions when CCRL2 or ChemR23 were depleted (Fig. 8a). We next explored the long-term effects of depleting CCRL2 and ChemR23 on chemerin-induced neural events. The analysis showed that the proportion of NeuN-positive adult-born neurons decreased in the olfactory bulb and hippocampal dentate gyrus of 2-month-old offspring from chemerin-induced diabetic dams compared to the control group, whereas the expression of NeuN-positive cells was rescued in the absence of CCRL2 or ChemR23, suggesting that removing CCRL2 and ChemR23 resulted in a long-term neuroprotective effect (Fig. 8b).

**Fig. 9:**
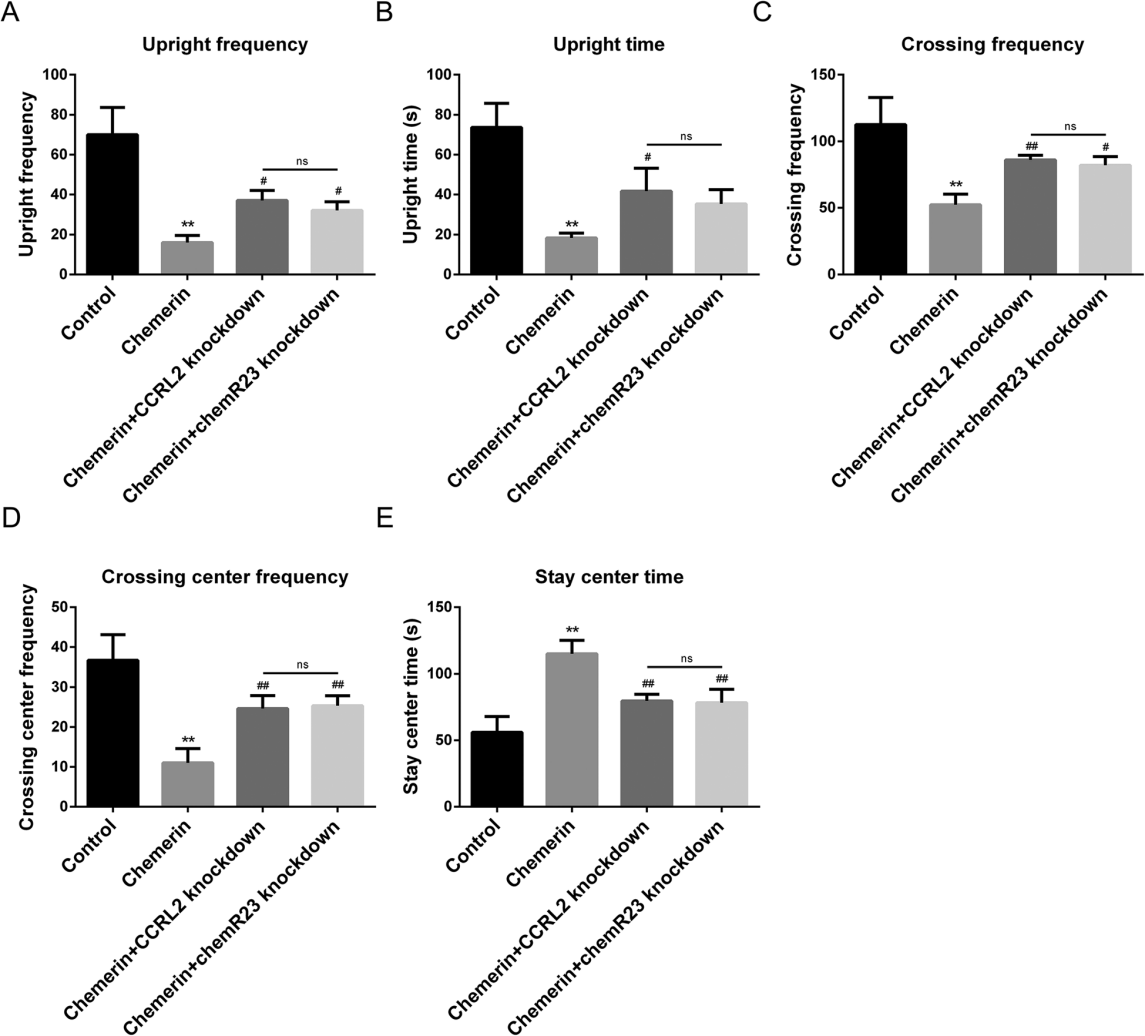
Effects of CCRL2 and chemR23 removal on recognition memory of offspring of diabetic dams. Detection of rearing frequency (**a**) and rearing times (**b**) of 8-week-old offspring of a normal pregnancy and of chemerin-induced diabetic dams, chemerin-induced diabetic dams with CCRL2-knockdown, and chemerin-induced diabetic dams with ChemR23-knockdown. Measurement of crossing frequency between squares (**c**) and frequency of crossing the center squares (**d**) by 8-week old offspring. **e** Comparative analysis of immobility time in 8-week old offspring. *Chemerin-induced diabetic dams vs. controls, # chemerin-induced diabetic dams with ChemR23 knockdown, and chemerin-induced diabetic dams with CCRL2 knockdown vs. chemerin-induced diabetic dams. ns, not significant; * and #—*P <* 0.05; ** and ##—*P <* 0.01

We observed the same abnormal response in the OFT assay of 8-week-old offspring as shown in Fig. 3 for the chemerin-induced maternal diabetes group, including the decrease in rearing time, rearing frequency, crossing frequency between squares, frequency of crossing the center squares, and the increase in immobility time (Fig. 9). The changes in horizontal and vertical activity in the offspring from chemerin-induced diabetic group were reversed after ChemR23 or CCRL2 knockdown. Rearing times, rearing frequency, and crossing frequency between squares and frequency of crossing the center squares improved compared to offspring from the chemerin-induced diabetic group, and the time remaining in the center decreased in the offspring from the diabetic group with ChemR23- and CCRL2-knockdown. And there was no significant difference between the two groups in the comparisons of the five indicators (Fig. 9a–e).

## Discussion

In this report, we proposed and verified our hypothesis that chemerin is enriched by CCRL2 in chemerin-induced diabetic dams’ offspring brain tissue, and chemerin aggregation in the offspring brain induced a decrease in neurons, accumulation of macrophages, and activation of pyroptosis in macrophages in a ChemR23-dependent manner, thereby leading to nerve damage and cognitive deficits in the offspring. Our results indicate that removing CCRL2 and/or ChemR23 could block some parts of this pathway and attenuate memory recognition impairment in offspring.

Neuropsychological deficits in offspring of diabetic mothers have been observed for decades. Although maternal inflammatory factors have been confirmed as pivotal in the induction of such deficits, the mechanism has not been revealed. Herein, we showed an association of the adipocytokine chemerin, which is involved in metabolism and inflammation [31–35], with neuropsychological deficits in chemerin-induced diabetic dams’ offspring.

Chemerin is expressed at extremely low levels in brain tissue and several types of cells [36, 37], similar to what we observed in the offspring of controls in the present study. However, chemerin aggregated in the brain tissue of offspring of mice with diabetic pregnancy. CCRL2 might be the key to the aggregation of chemerin in brain tissue. In fact, CCRL2 is expressed in a variety of brain cells, such as astrocytes, glial cells, and brain endothelial cells [38, 39]. Neither Ca^2+^ mobilization nor internalization were detected after incubating chemerin with CCRL2, due to a lack of intracellular signaling [40]. However, binding of CCRL2 to chemerin promotes local concentrations of bioactive chemerin [24]. Additionally, there is evidence that CCRL2-chemerin-ChemR23^+^ cell recruitment/inflammation signaling transduction participates in the biological processes of brain endothelial cells [33, 41]. Similar to these studies, we found that the interaction between chemerin and CCRL2 was enhanced in the brain of offspring of diabetic mice. Depleting CCRL2 resulted in a decrease of chemerin in brain tissues, suggesting that CCRL2 might be the key element involved in the enrichment of chemerin in brain tissues of offspring of diabetic dams.

As a chemokine, chemerin has been known to be responsible for the recruitment of macrophages for more than 10 years [42]. However, whether the accumulation of chemerin promotes invasion by macrophages in the brain tissue, and even causes abnormal behavior, has not been elucidated. A higher level of chemerin accompanied by more macrophages, and a subsequent increase in inflammation and apoptosis-associated molecules (NLRP3 and Asc) in the brain tissues of offspring of diabetic dams, was observed along with aberrant recognition memory in 8-week-old offspring; these findings indicate that chemerin-macrophage enrichment in brain tissue may participate in the development of brain diseases. Chemerin recruits macrophages in vivo and in vitro [15, 16], but we first demonstrated macrophage recruitment by chemerin in the brain tissue of offspring from diabetic dams, which was associated with brain injury. We also found chemerin-induced activation of pyroptosis in macrophages, but not of the apoptosis pathway, followed by the release of inflammation cytokines and an increase in NLRP3. Ex vivo and in vitro studies show that chemerin recruits macrophages by binding to ChemR23 [15, 42]. ChemR23-knockdown in diabetic mice resulted in reduced macrophage invasiveness, activation of pyroptosis, and subsequent secretion of inflammatory factors into the fetal brain, demonstrating that chemerin recruits macrophages into the brain tissue in a ChemR23-dependent manner. In addition, chemerin administration induced the recruitment of ChemR23 in macrophages rather than neurons. Moreover, chemerin exposure had no toxic effect on nerve cells. Since the toxic effect of chemerin on the brain depends on the expression distribution of ChemR23, it would not develop directly toxic effects on neurons because chemR23 is a weak expression in nerve cells.

Some limitations of our study should be discussed. First, the mechanism of chemerin-mediated neuropsychological deficits in the offspring was explored only in chemerin-induced diabetic model; ideally, other models, especially gestational diabetes mellitus model (according to its high prevalence), should also be employed for comprehensively understanding the potential mechanism of maternal diabetic offspring’s brain impairment. However, there is no ideal animal model completely mimicking diabetes in pregnancy, which makes it difficult to fully understand the association between maternal diabetes and offspring’s neuropsychological deficits. Thus, it needs more study to reveal the underlying specific mechanism of complication from maternal diabetes and to gradually move the field of diabetic pregnancy forward from bench to bedside. Second, we did not study how activation of ChemR23 induces activation of the NLRP3 inflammasome/pyroptosis in macrophages. In fact, the chemerin-ChemR23 interaction mainly mobilizes intracellular Ca2+ [40], which is an essential part of activating the NLRP3 inflammasome [43]; further studies are needed.

## Conclusions

In summary, administering chemerin induced the maternal diabetic phenotype in mouse dams, followed by aggregation of chemerin in the presence of CCRL2 and macrophages’ recruitment by ChemR23 in the fetal mice brain, leading to brain inflammation, subsequent neuronal injury, and abnormal cognitive performance of offspring. Pregnant diabetes-induced inflammatory response during embryogenesis could interfere with the development of neuronal circuitry, which would negatively affect cognitive development in the offspring. Our results will contribute to the prevention of the inflammatory damage-mediated impairment in recognition memory in offspring brains.

## Additional files

Additional file 1: **Figure S1.** Effects of chemerin on cortex, mature neurons and the concentration of chemerin in brain tissue. (A) Integrated optical density per unit area (IOD/area) of β-III-tubulin level in coronal cortical sections at E18.5 and NeuN level in Olfactory bulb and dentate gyrus of 8-week-old offspring from controls and chemerin-induced diabetic group. (B) The concentration of chemerin measured by ELISA kit in brain tissue of E18.5 and 7-day-old offspring from chemerin-induced diabetic dams. *chemerin or chemerin+CCRL2-knockdown vs control; #chemerin+CCRL2-knockdown vs chemerin. #, *P <* 0.05; ** and ##, *P <* 0.01. (PDF 1005 kb)

Additional file 2: **Figure S2.** Immunofluorescence staining results. **(A)** CCRL2 and ChemR23 knockdown efficiency in 18.5-day-old fetal mice and 7-day-old offspring from diabetic dams and macrophages isolated from the peritoneal cavity of mice. **(B)** IOD/area of chemerin- and CCRL2-positive cells in forebrain tissues of 18.5-day-old fetal mice and 7-day-old offspring from controls, chemerin-induced diabetic dams, and chemerin-induced diabetic dams with ChemR23 knockdown mice. **(C)** IOD/area of F4/80- and MAP2-positive cells. **(D)** IOD/area of chemerin- and F4/80-positive cells. **(E)** IOD/area of β-III-tubulin level in coronal cortical sections at E18.5 and NeuN level in Olfactory bulb and dentate gyrus of 8-week-old offspring from controls, chemerin-induced diabetic dams, chemerin-induced diabetic dams with ChemR23 knockdown and chemerin-induced diabetic dams with CCRL2 knockdown mice. * chemerin-induced diabetic dams vs. controls (E18.5d); #chemerin-induced diabetic dams with ChemR23 knockdown/CCRL2 knockdown vs. chemerin-induced diabetic dams (E18.5d); ^GDM group vs. controls (B7.0d); $chemerin-induced diabetic dams with ChemR23 knockdown/CCRL2 knockdown vs. chemerin-induced diabetic dams (B7.0d). * and #, *P <* 0.05; **, ##, ^^ and $$, *P <* 0.01. (PDF 5692 kb)

Additional file 3: **Figure S3.** FACS sorting for macrophages and effect of chemerin on migration of macrophages**. (A)** Macrophages, microglia, and other cell fractions were sorted by FACS from a pool of mononuclear cells isolated from brain tissues of 18.5-day-old fetal mice. CD45 high, CD11b high, F4/80 high, and Gr-1 low indicate the macrophage fraction; CD45 intermediate and CD11b intermediate indicate the microglial fraction; and CD11b negative and Gr-1 high indicates other cell fractions. **(B)** Levels of ChemR23 were detected by western blotting in a pool of macrophages isolated from the peritoneal cavity of normal mice. Macrophages were stimulated with 0, 1, 10, 100, or 1000 nM chemerin for 30 min. The histogram represents the gray values of bands normalized to GAPDH. **(C)** The proportion of migrated macrophages was measured by Transwell assay. CXCL8 treatment was defined as the positive control. Data are presented as mean with 95% CI. * chemerin treatment vs. control. **P < 0.01. (PDF 4414 kb)

Additional file 4: **Figure S4.** Analysis of toxic effects of chemerin on neurons**. (A)** The expression and distribution of ChemR23, F4/80 and MAP2 in brain tissue sections of E18.5 and 7-day-old offspring as analyzed by immunofluorescent staining. DAPI: blue; ChemR23: red; F4/80: green; MAP2: gray. Scale bar: 50 μm. **(B)** After exposed with 1, 5 and 10 nm chemerin, Apoptosis of primary neurons as evaluated by TUNEL staining. DAPI: blue; TUNEL-positive cells: green. (PDF 10479 kb)

## Data Availability

All data generated or analyzed during this study are included in this published article.

## Abbreviations

Asc: Apoptosis-associated speck-like protein
BBB: Blood-brain barrier
BEB: Blood-embryo barrier
CCRL2: Chemokine (C-C motif) receptor-like 2
ChemR23: Chemerin receptor 23
CI: Confidence interval
CP: Cortical plate
Ct: Mean cycle threshold
ELISA: Enzyme-linked immunosorbent assay
FACS: Fluorescence-activated cell sorting
FBG: Fasting blood glucose
GAPDH: Glyceraldehyde-3-phosphate dehydrogenase
GD: Gestational day
IL: Interleukin
IOD/Area: Integrated optical density per unit area
IZ: Intermediate zone
LDH: Lactate dehydrogenase
MAP 2: Microtubule-associated protein 2
NeuN: Neuronal nuclear antigen
NLRP3: NOD-like receptor family pyrin domain containing 3
OF: Open field
OFT: Open field test
OGTT: Oral glucose tolerance test
PBS: Phosphate-buffered saline
PCR: Polymerase chain reaction
pfu: Plaque-forming units
shRNA: Short hairpin RNA
STZ: Streptozocin
VZ/SVZ: Ventricular and subventricular precursor zones
