# Supplementary figures and images for: Chemerin-induced macrophages pyroptosis in fetal brain tissue leads to cognitive disorder in offspring of diabetic dams
Source: J Neuroinflammation. 2019 Nov 16;16:226. doi: 10.1186/s12974-019-1573-6 (PMC6858779; doi:10.1186/s12974-019-1573-6)

A

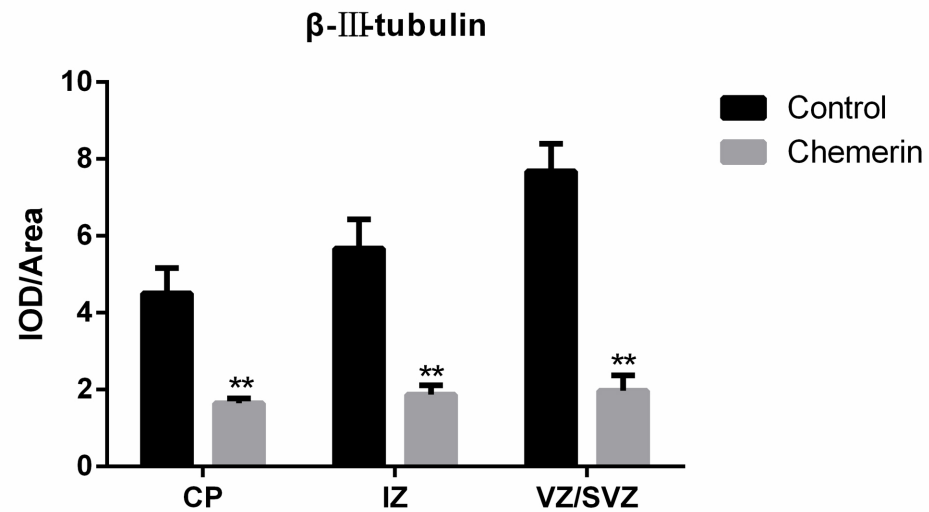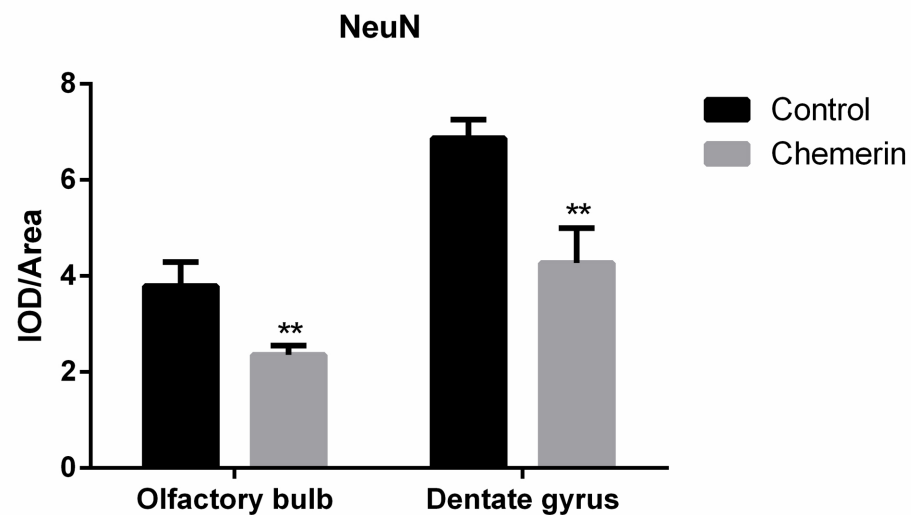

B

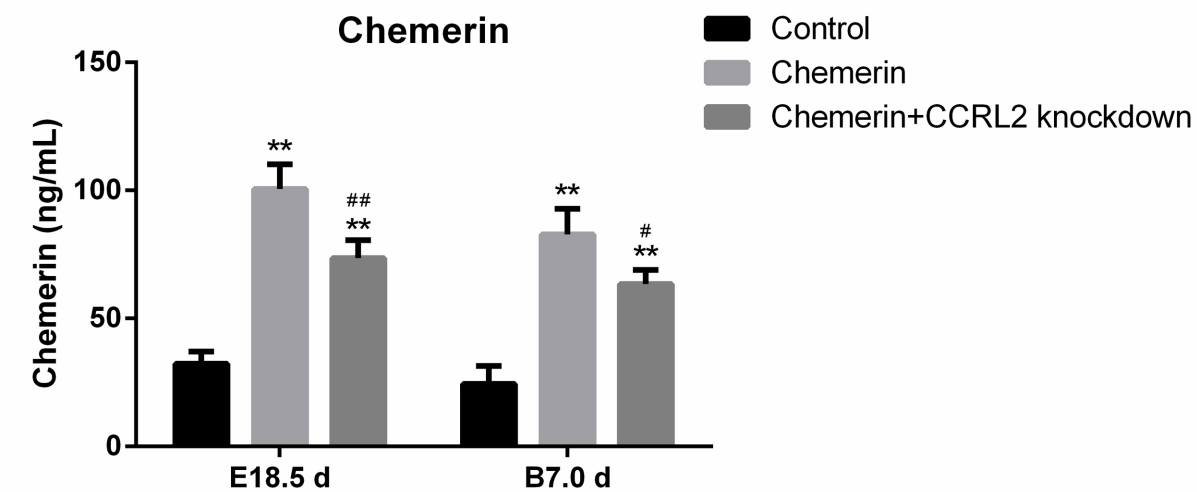

Supplement: Supplementary file 1 — Figure S1. Effects of chemerin on cortex, mature neurons and the concentration of chemerin in brain tissue. (A) Integrated optical density per unit area (IOD/area) of β-III-tubulin level in coronal cortical sections at E18.5 and NeuN level in Olfactory bulb and dentate gyrus of 8-week-old offspring from controls and chemerin-induced diabetic group. (B) The concentration of chemerin measured by ELISA kit in brain tissue of E18.5 and 7-day-old offspring from chemerin-induced diabetic dams. *chemerin or chemerin+CCRL2-knockdown vs control; #chemerin+CCRL2-knockdown vs chemerin. #, P < 0.05; ** and ##, P < 0.01. (PDF 1005 kb) [file 12974_2019_1573_MOESM1_ESM.pdf]

A

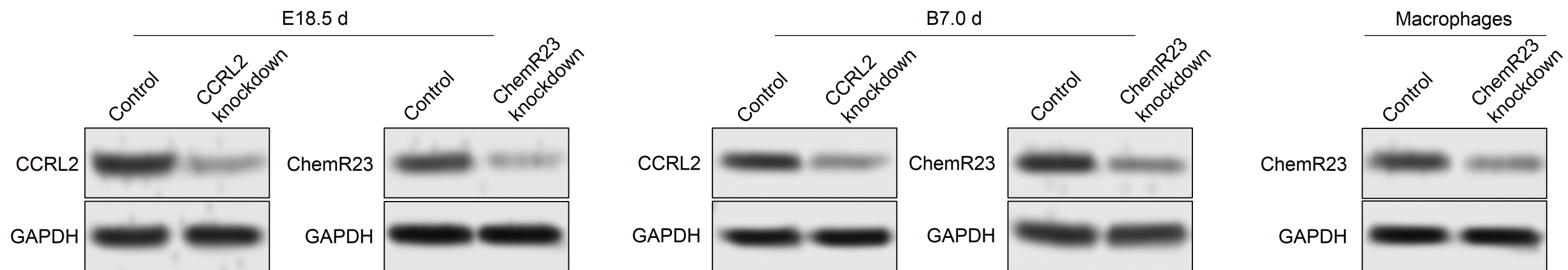

B

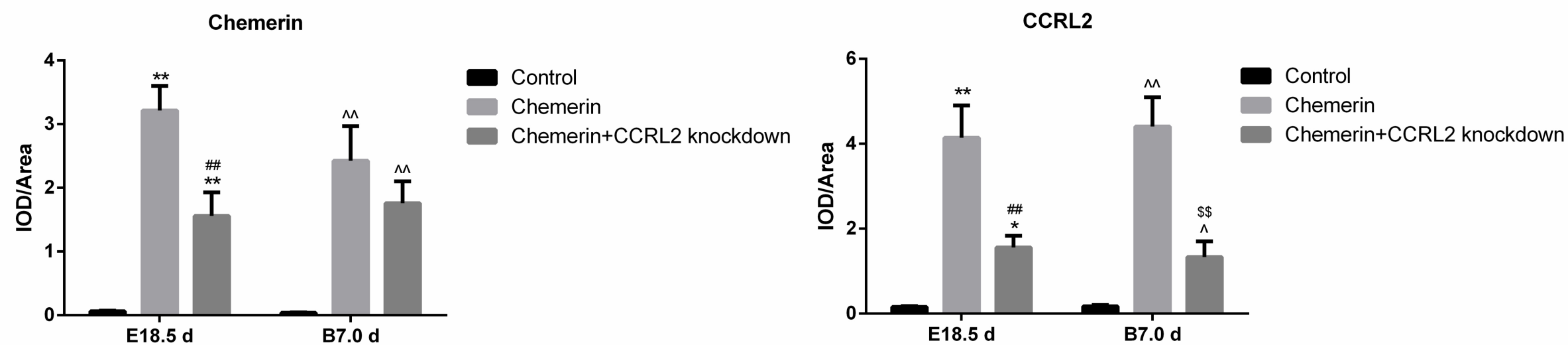

C

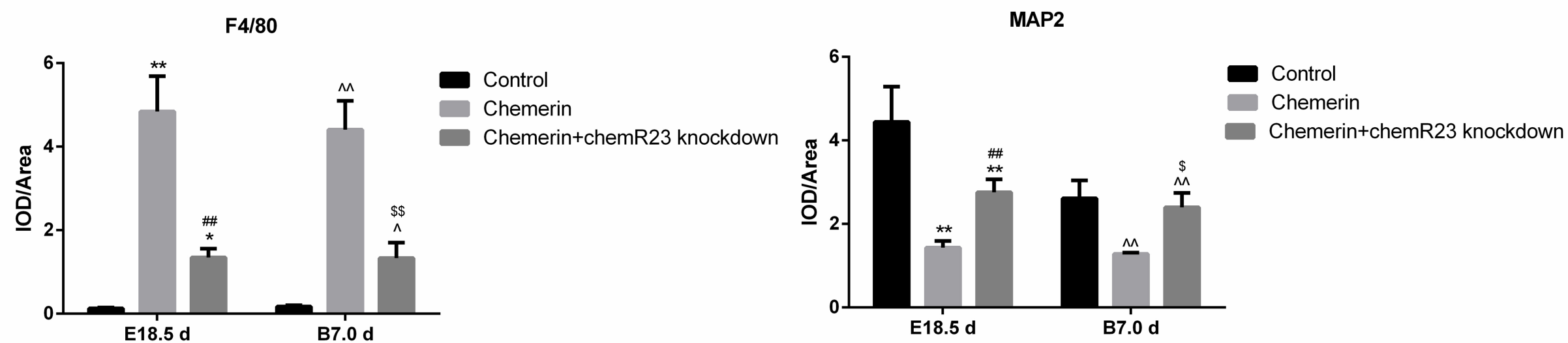

D

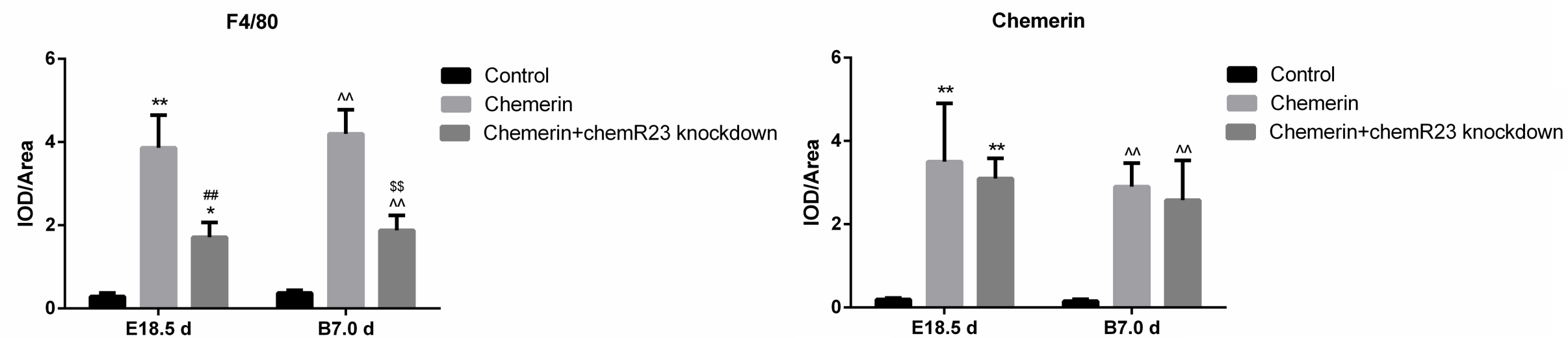

E

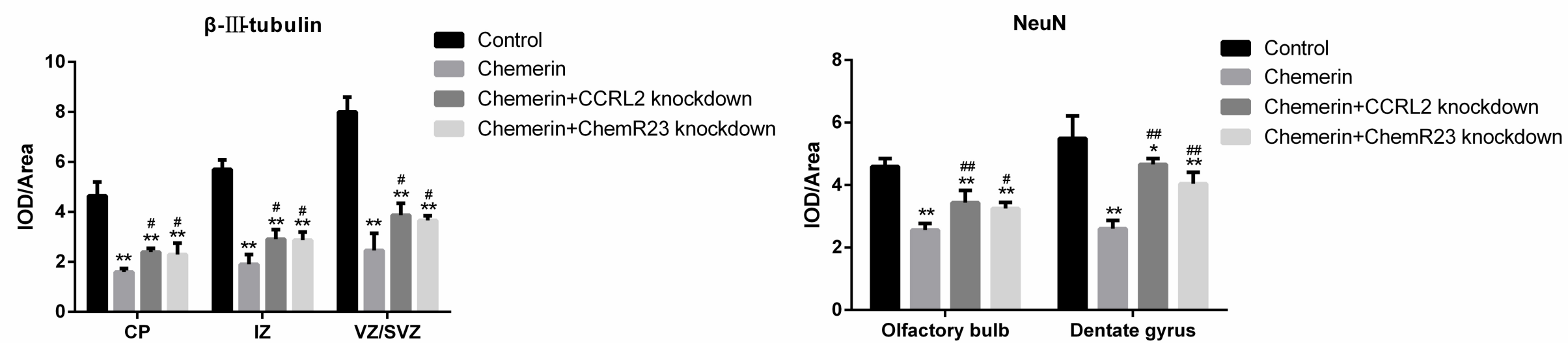

Supplement: Supplementary file 2 — Figure S2. Immunofluorescence staining results. (A) CCRL2 and ChemR23 knockdown efficiency in 18.5-day-old fetal mice and 7-day-old offspring from diabetic dams and macrophages isolated from the peritoneal cavity of mice. (B) IOD/area of chemerin- and CCRL2-positive cells in forebrain tissues of 18.5-day-old fetal mice and 7-day-old offspring from controls, chemerin-induced diabetic dams, and chemerin-induced diabetic dams with ChemR23 knockdown mice. (C) IOD/area of F4/80- and MAP2-positive cells. (D) IOD/area of chemerin- and F4/80-positive cells. (E) IOD/area of β-III-tubulin level in coronal cortical sections at E18.5 and NeuN level in Olfactory bulb and dentate gyrus of 8-week-old offspring from controls, chemerin-induced diabetic dams, chemerin-induced diabetic dams with ChemR23 knockdown and chemerin-induced diabetic dams with CCRL2 knockdown mice. * chemerin-induced diabetic dams vs. controls (E18.5d); #chemerin-induced diabetic dams with ChemR23 knockdown/CCRL2 knockdown vs. chemerin-induced diabetic dams (E18.5d); ^GDM group vs. controls (B7.0d); $chemerin-induced diabetic dams with ChemR23 knockdown/CCRL2 knockdown vs. chemerin-induced diabetic dams (B7.0d). * and #, P < 0.05; **, ##, ^^ and $$, P < 0.01. (PDF 5692 kb) [file 12974_2019_1573_MOESM2_ESM.pdf]

A

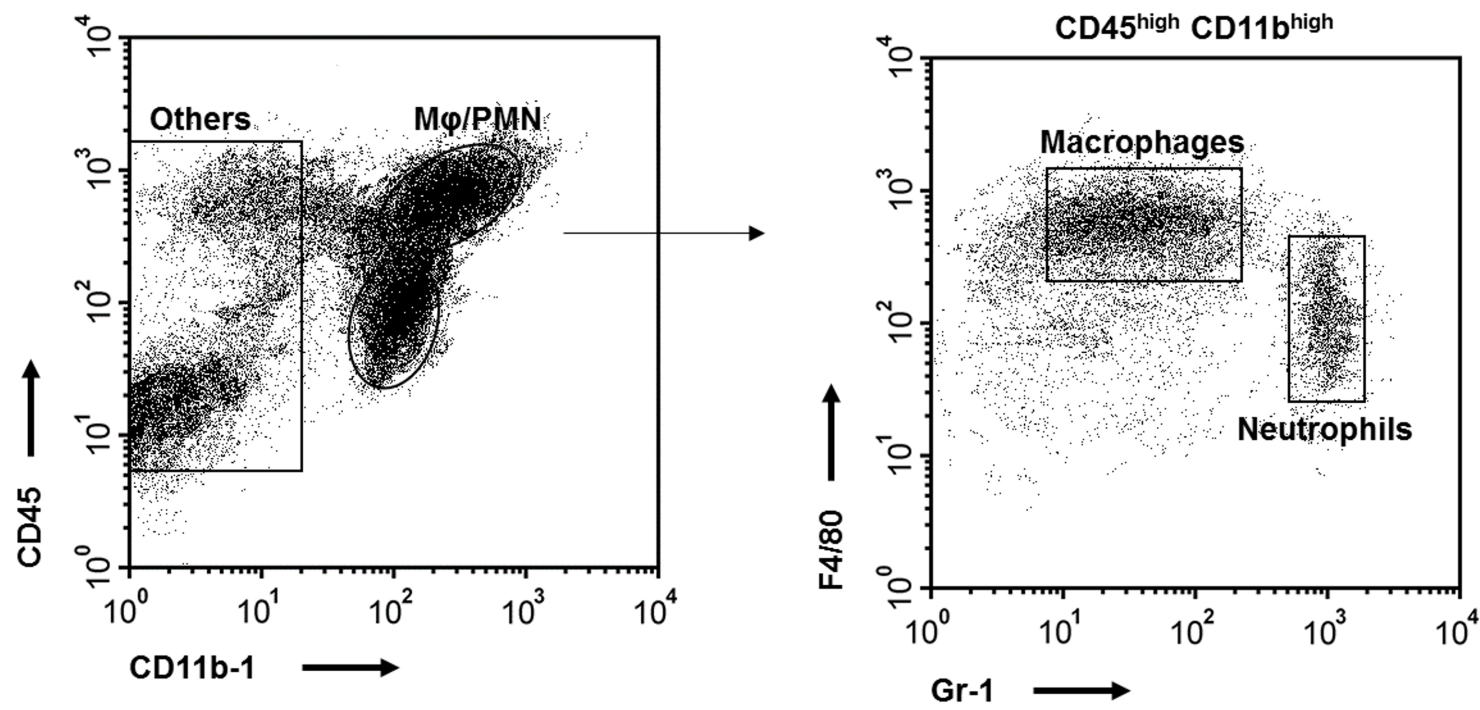

B

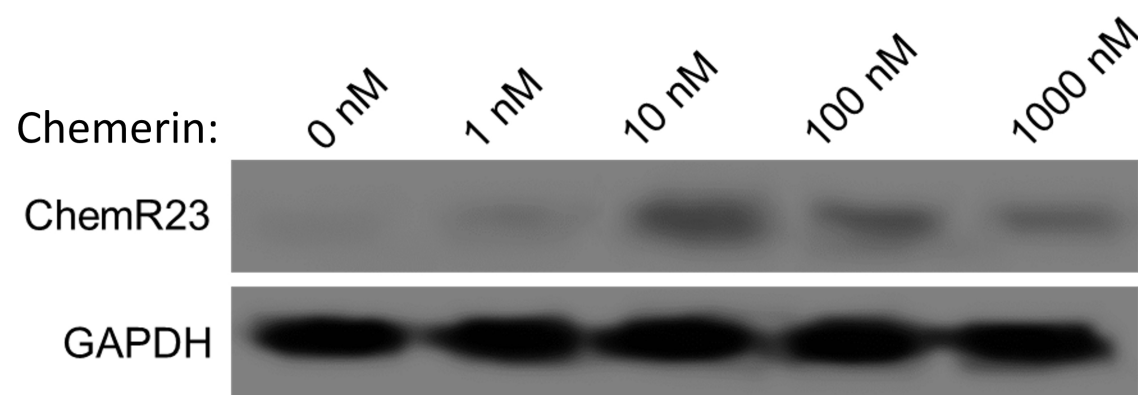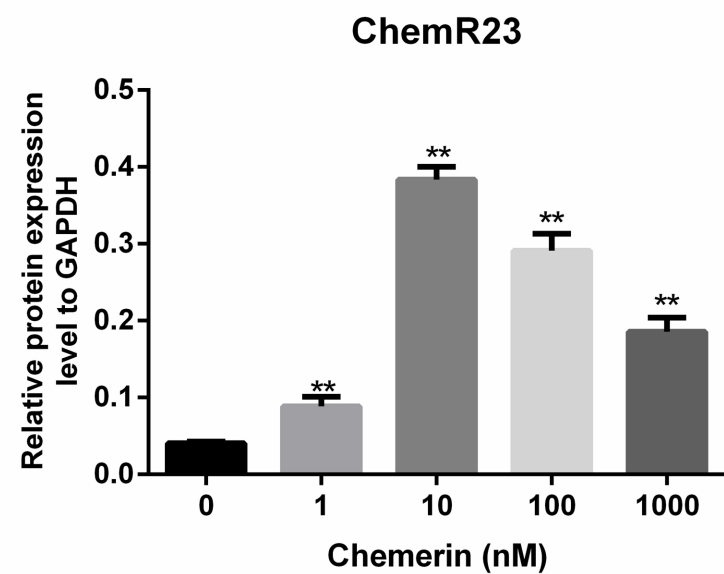

C

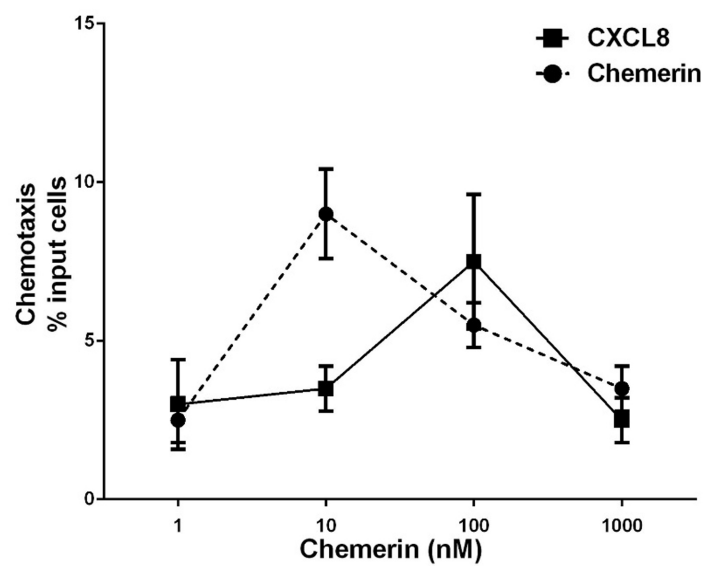

Supplement: Supplementary file 3 — Figure S3. FACS sorting for macrophages and effect of chemerin on migration of macrophages. (A) Macrophages, microglia, and other cell fractions were sorted by FACS from a pool of mononuclear cells isolated from brain tissues of 18.5-day-old fetal mice. CD45 high, CD11b high, F4/80 high, and Gr-1 low indicate the macrophage fraction; CD45 intermediate and CD11b intermediate indicate the microglial fraction; and CD11b negative and Gr-1 high indicates other cell fractions. (B) Levels of ChemR23 were detected by western blotting in a pool of macrophages isolated from the peritoneal cavity of normal mice. Macrophages were stimulated with 0, 1, 10, 100, or 1000 nM chemerin for 30 min. The histogram represents the gray values of bands normalized to GAPDH. (C) The proportion of migrated macrophages was measured by Transwell assay. CXCL8 treatment was defined as the positive control. Data are presented as mean with 95% CI. * chemerin treatment vs. control. **P < 0.01. (PDF 4414 kb) [file 12974_2019_1573_MOESM3_ESM.pdf]

# A

E18.5 d

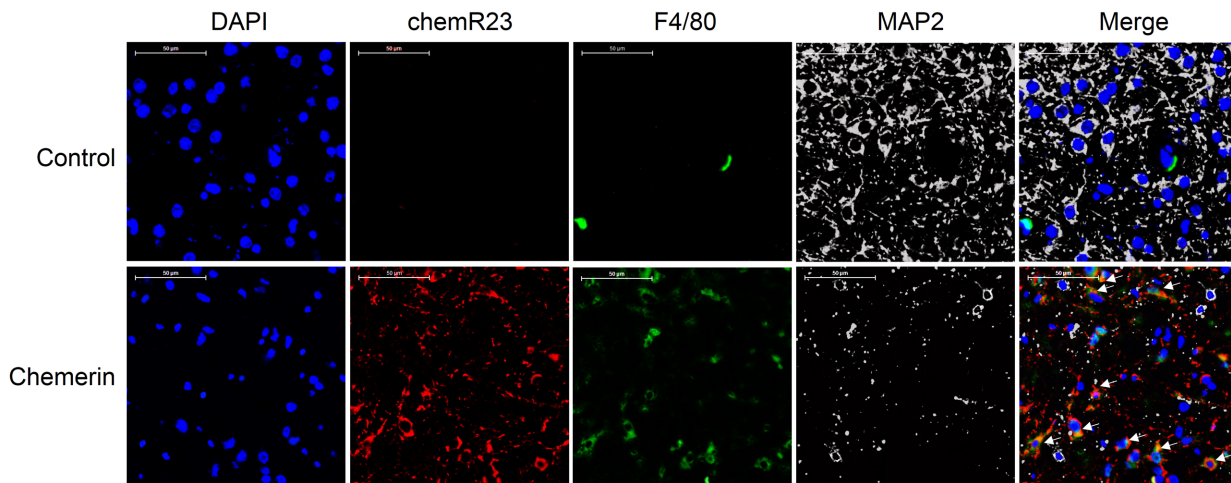

D7.0 d

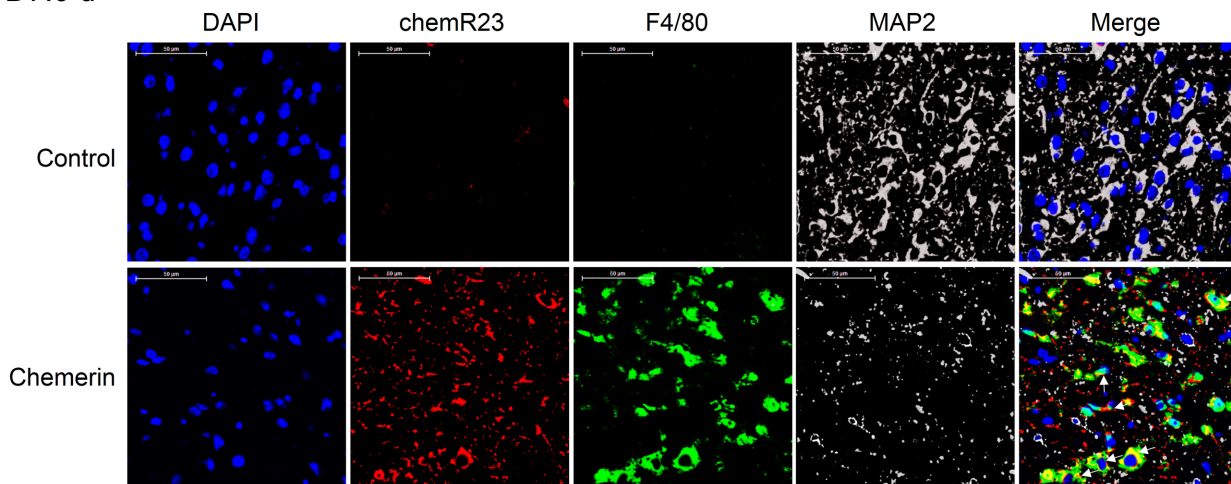

# B

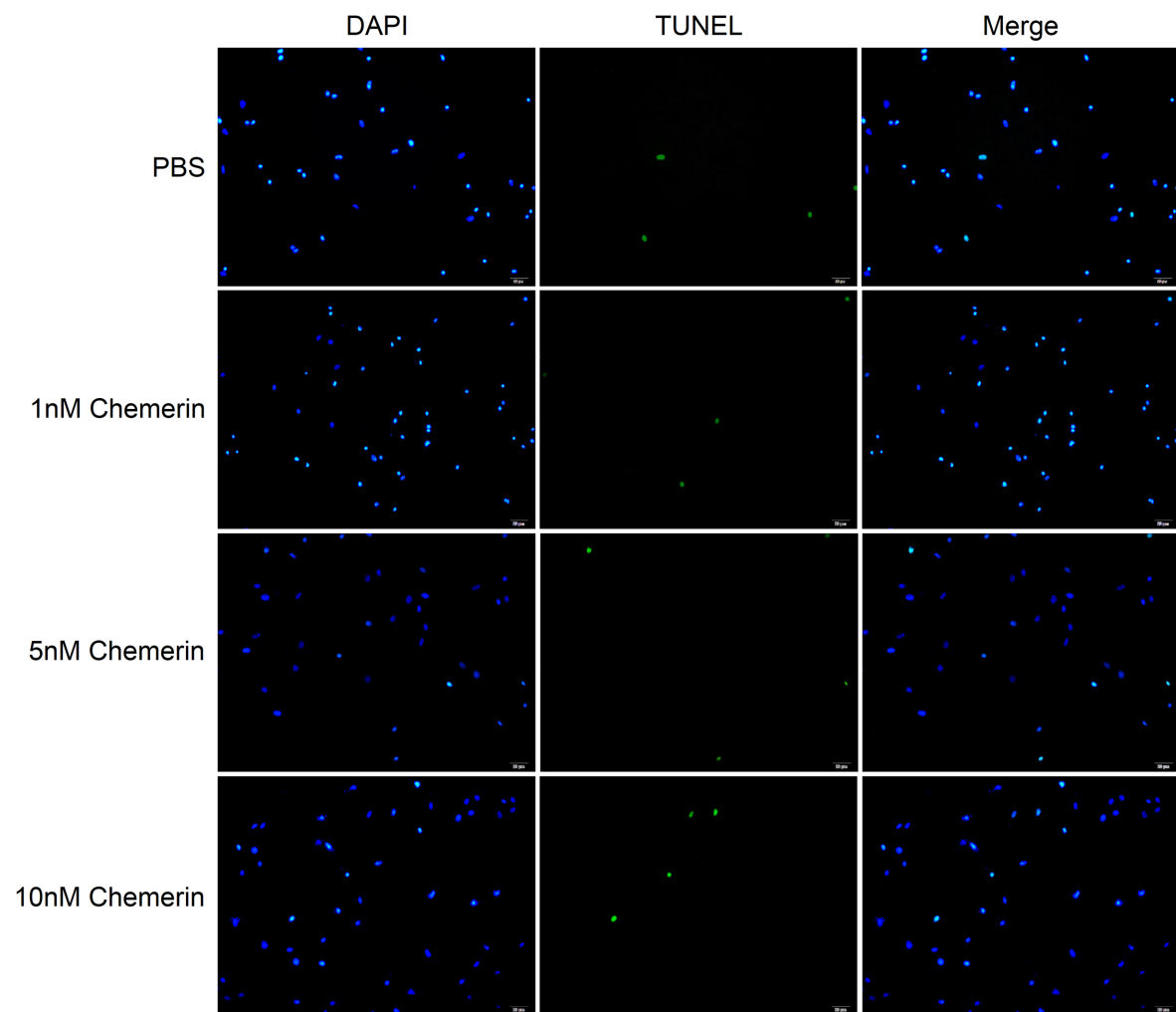

Supplement: Supplementary file 4 — Figure S4. Analysis of toxic effects of chemerin on neurons. (A) The expression and distribution of ChemR23, F4/80 and MAP2 in brain tissue sections of E18.5 and 7-day-old offspring as analyzed by immunofluorescent staining. DAPI: blue; ChemR23: red; F4/80: green; MAP2: gray. Scale bar: 50 μm. (B) After exposed with 1, 5 and 10 nm chemerin, Apoptosis of primary neurons as evaluated by TUNEL staining. DAPI: blue; TUNEL-positive cells: green. (PDF 10479 kb) [file 12974_2019_1573_MOESM4_ESM.pdf]
